# IKKα plays a major role in canonical NF-κB signalling in colorectal cells

**DOI:** 10.1042/BCJ20210783

**Published:** 2022-02-04

**Authors:** Jack A. Prescott, Kathryn Balmanno, Jennifer P. Mitchell, Hanneke Okkenhaug, Simon J. Cook

**Affiliations:** 1 Signalling Programme, The Babraham Institute, Babraham Research Campus, Cambridge CB22 3AT, U.K.; 2 Imaging Facility, The Babraham Institute, Babraham Research Campus, Cambridge CB22 3AT, U.K.

**Keywords:** colorectal cancer cells, IKKβ, IL-1, NF-κB, TNF

## Abstract

Inhibitor of kappa B (IκB) kinase β (IKKβ) has long been viewed as the dominant IKK in the canonical nuclear factor-κB (NF-κB) signalling pathway, with IKKα being more important in non-canonical NF-κB activation. Here we have investigated the role of IKKα and IKKβ in canonical NF-κB activation in colorectal cells using CRISPR–Cas9 knock-out cell lines, siRNA and selective IKKβ inhibitors. IKKα and IKKβ were redundant for IκBα phosphorylation and turnover since loss of IKKα or IKKβ alone had little (SW620 cells) or no (HCT116 cells) effect. However, in HCT116 cells IKKα was the dominant IKK required for basal phosphorylation of p65 at S536, stimulated phosphorylation of p65 at S468, nuclear translocation of p65 and the NF-κB-dependent transcriptional response to both TNFα and IL-1α. In these cells, IKKβ was far less efficient at compensating for the loss of IKKα than IKKα was able to compensate for the loss of IKKβ. This was confirmed when siRNA was used to knock-down the non-targeted kinase in single KO cells. Critically, the selective IKKβ inhibitor BIX02514 confirmed these observations in WT cells and similar results were seen in SW620 cells. Notably, whilst IKKα loss strongly inhibited TNFα-dependent p65 nuclear translocation, IKKα and IKKβ contributed equally to c-Rel nuclear translocation indicating that different NF-κB subunits exhibit different dependencies on these IKKs. These results demonstrate a major role for IKKα in canonical NF-κB signalling in colorectal cells and may be relevant to efforts to design IKK inhibitors, which have focused largely on IKKβ to date.

## Introduction

The nuclear factor-κB (NF-κB) transcription factors (homo- and hetero-dimers of p65/RelA, RelB, c-Rel, NF-κB1/p50 and NF-κB2/p52) are sequestered in the cytoplasm through their binding to inhibitor of kappa B (IκB) proteins. In response to cytokines and stress signals, the IκB proteins are rapidly phosphorylated by the IκB kinases (IKKs) [1], triggering their proteasomal degradation and allowing NF-κB to enter the nucleus to drive gene expression. In the canonical NF-κB pathway tumour necrosis factor (TNFα) or interleukin-1 (IL-1) receptor signalling activates TAK1, which phosphorylates and activates IKKα and IKKβ when they are bound to dimers of a regulatory subunit, NEMO (NF-κB essential modifier).

Early studies revealed distinct and non-redundant functions for IKKα and IKKβ. Loss of IKKβ was embryonic lethal in mice [2,3] and IKKβ-null mouse embryonic fibroblasts (MEFs) exhibited striking defects in phosphorylation and degradation of IκBα and NF-κB activity following treatment with TNFα or IL-1 [4,5]. Similarly, conditional knockouts showed the importance of IKKβ in macrophages [6], osteoclasts [7] and the gut epithelium [8,9]. In contrast, IKKα-null mice survived a month post-birth, but developed defects in epidermal and skeletal development [3,10,11] that were independent of IKKα kinase activity and NF-κB [12]. A similar, more severe phenotype was seen in patients with autosomal recessive loss-of-function mutations in *CHUK* (encoding human IKKα) [13]. An essential role for IKKα in non-canonical NF-κB activation in developing lymphoid organs and B cell maturation was subsequently demonstrated [14–17]. Whilst IKKα-null MEFs showed some defects in p65 phosphorylation and NF-κB activity in response to TNFα or IL-1, IKKβ homodimers were sufficient for canonical IκBα phosphorylation and NF-κB activation [2,3,18]. Furthermore, binding between NEMO and IKKα was weaker than with IKKβ [19,20] and IKKβ was a more effective IκBα kinase *in vitro* [21].

From these studies, a consensus emerged that IKKβ is the critical NEMO-dependent IKK in the canonical NF-κB pathway, with IKKα acting in the noncanonical pathway, independent of NEMO. As such, IKKβ has been the focus of drug discovery efforts [22,23]; development of IKKα inhibitors has lagged behind, partly reflecting the difficulty in targeting the IKKα catalytic site [24]. Despite this consensus, reports continue to suggest IKKα is important in canonical NF-κB signalling. For example, TNFα- or IL-1-stimulated, NF-κB-dependent gene expression persists in IKKβ-null MEFs [2,4]. Furthermore, in contrast with the developing liver, mature hepatocytes are not sensitised to TNFα-induced apoptosis following IKKβ deletion, suggesting that IKKα can compensate in adult mice [25]. IKKα is also obligatory for RANK-induced canonical NF-κB activation in mammary epithelial cells [12]. In contrast with earlier studies, IL-1, but not TNFα, promotes NEMO-dependent IκBα degradation and NF-κB activation in IKKβ-null MEFs [26]. The same laboratory demonstrated that TNF-α or IL-1 induced NF-κB activity was completely defective in IKKα-null MEFs and suggested IKKα activates canonical NF-κB independently of NEMO [27], possibly through direct phosphorylation of p65.

In contrast with MEFs, both IKKα and IKKβ contribute to TNFα-induced NF-κB activation in HeLa cells [28]. Furthermore, patients with homozygous null mutations of *IKBKB* exhibit less severe phenotypes than IKKβ-null mice [29,30]; whilst they go on to develop severe combined immunodeficiency, they are normal at birth and exhibit no liver damage or developmental defects seen in IKKβ-null mice, suggesting that IKKα can compensate within the canonical NF-κB pathway in human cells. Finally, whilst primary skin fibroblasts from these patients exhibited drastically reduced NF-κB activation in response to TNFα; the response to IL-1β was marginally affected [29]. Overall, it seems the role of IKKα and IKKβ in canonical NF-κB signalling varies with cell- or tissue-type, developmental stage, stimulus and even species, making it critical to assess the role of the IKKs in physiologically and pathologically relevant systems rather than relying on studies in MEFs.

We are interested in activation of NF-κB and inflammation in the colon where inflammatory bowel disease (IBD) incidence increases with age and may predispose to cancer. This is well established in colorectal cancer (CRC) where NF-κB promotes progression of ulcerative colitis and Crohn's disease, to colitis-associated cancer (CAC) [31]. Anti-TNFα therapy is employed in both colitis and Crohn's and NF-κB is constitutively active in human tumour samples from both CAC and sporadic CRC and is associated with poor prognosis [32–34]. Constitutive NF-κB activation is also observed in CRC cell lines, suggesting that cell-autonomous mechanisms may lead to aberrant pathway activation, in addition to inflammatory conditions within the tumour microenvironment [35,36]. Indeed, intestinal epithelial cell-specific deletion of IKKβ decreased tumour incidence in a mouse model of CAC [8]. Given the importance of inflammatory NF-κB signalling in IBD and CRC and the debate on the relative role of IKKα and IKKβ and we generated IKKα, IKKβ and IKKα/β double knockouts in colorectal cancer cell lines. Here, our initial characterisation of these cells reveals an unexpected and prominent role for IKKα in canonical NF-κB signalling.

## Materials and methods

### Cell culture, transfections and plasmids

HCT116 cells were cultured at 37°C in a humidified incubator with 95% air and 5% CO_2_ in DMEM (Life Technologies) supplemented with 10% (v/v) foetal bovine serum, 2 mM L-glutamine, 100 U/ml penicillin and 100 µg/ml streptomycin. Cells were passaged at ∼80% confluence every 3–4 days by enzymatic detachment with 0.05% trypsin/EDTA solution. SW620 cell were cultured at 37°C in a humidified incubator with 95% air and 5% CO_2_ in Liebowitz's L-15 media (Life Technologies) also supplemented with 10% (v/v) foetal bovine serum, 2 mM L-glutamine, 100 U/ml penicillin, 100 µg/ml streptomycin and 0.075% (v/v) sodium bicarbonate. Cells were passaged at ∼80% confluence every 3–4 days by enzymatic detachment with 0.05% trypsin/EDTA solution. Transfections were performed using Lipofectamine 2000 according to the manufacturer's instructions. pGL4.32[luc2P/NF-кB-RE was a kind gift from Prof. Neil Perkins (Institute for Cell and Molecular Biosciences, University of Newcastle, U.K.). *Wild-type* FLAG-IKKα and kinase inactive FLAG-IKKα were kind gifts from Prof. Lienhard Schmitz (Department of Biochemistry, Justus-Liebig-Universität Gießen, Germany). *Wild-type* pCMV2TAG-IKKβ and kinase inactive pCMV2TAG-IKKβ (K44M) were purchased from Addgene (Plasmid #11103 and 11104, respectively). pD1301-AD Cas9 expression vector was supplied by Horizon Discovery™.

### RT-qPCR gene expression analysis

Total RNA was extracted from cells using the RNeasy Kit (QIAGEN), QIAshredders (QIAGEN) and RNase-Free DNase Set (QIAGEN) according to manufacturer's instructions. An amount of 1 µg RNA was reverse transcribed to cDNA using the Quantitech Reverse Transcription kit according to manufacturer's instructions. cDNA was diluted 1 : 20 in RNase-free water and stored at −20°C. RT-qPCR was performed using SYBR Green (QIAGEN) and primers with the following sequences: IKKα: F 5′-TGTACACAGAGTTCTGCCCG-3′, R 5′-TGAAGTCTCCCCATCTTGAGG 3′; IKKβ: F 5′-CTTGGGACAGGGGGATTTGG-3′, R 5′-ATTGGGGTGGGTCAGCCTTC-3′; YWHAZ: F 5′-TGATCCCCAATGCTTCACAAG-3′, R 5′-GCCAAGTAACGGTAGTAATCTCC-3′. This was performed in a 96-well plate format using a CFX96 Real-Time PCR Detection System (Bio-Rad). Data were normalised to the geometric mean of the reference gene expression and relative gene expression change calculated using the Pfaffl method. Statistical analysis was performed with the REST 2009 software package using an applied two-sided pair-wise fixed reallocation randomisation test.

### CRISPR genome editing

Target guide RNA (gRNA) sequences targeting either *CHUK* (IKKα) or *IKBKB* (IKKβ) genes were designed using the gUIDEbook™ software (Desktop Genetics). The sequences used to target *CHUK* were 5′-AGCCGCTCCCGCATCTCCCA-3′, 5′-ACAGACGTTCCCGAAGCCGC-3′ and 5′-TCTTCATAATCTGGATTTCA-3′, and for *IKBKB*, 5′-AGCCGCTCCCGCATCTCCCA-3′, 5′-TGTACCAGCATCGGGTGAGG-3′ and 5′-AGGCCTTTACAACATTGGC-3′. The sequences were cloned into pD1301-AD mammalian Cas9 vector containing a GFP reporter. HCT116 or SW620 cells were transfected by electroporation with 3.75 µg of either the IKKα-targeted Cas9 plasmid DNA, the IKKβ-targeted Cas9 plasmid DNA or empty-vector GFP Cas9 plasmid DNA before being combined and incubated at 37°C, 5% (v/v) CO_2_ for 48 h. For the double knockout of IKKα and IKKβ, 1.875 µg of each plasmid was transfected. Electroporation was performed with Nucleofector™ Kit V (Lonza) using programme D-032 on a Nucleofector™ device (Lonza). After 48 h, cells were harvested by trypsinisation, centrifuged and resuspended in 2% (v/v) FBS in PBS. The cell suspension was filtered through a 40 µm cell strainer (CellTrics). GFP-positive cells were single-cell sorted into 96-well plates containing 100 µl complete growth media containing 25% conditioned media using a FACS Aria III (BD) configured with an 85 µm nozzle. Single cell colonies were allowed to grow over a 10–14-day period and media replaced with fresh every 4 days. Upon confluency, cells were split into two 96-well plates: one for genotyping and one for clonal expansion. Genomic DNA was extracted from cells as described previously and used as a template to PCR amplify a 1 kb region spanning the gRNA target using Phusion High-Fidelity DNA polymerase (Invitrogen) according to manufacturer's instructions. Purified PCR product was cloned into pCR2.1 TOPO vector and transformed into XL1 Blue competent cells (Agilent). Multiple individual bacterial colonies were picked and sent for Sanger sequencing. Candidate clones were expanded and knockout was verified by Western blot.

### Western blot analysis

Whole-cell lysates were generated from cells suspended in tris-glycine (TG) lysis buffer containing 20 mM Tris–HCl (pH 7.5), 137 mM NaCl, 1 mM EGTA, 1% v/v Triton X-100, 10% v/v glycerol and 1.5 mM MgCl_2_ and freshly supplemented with 1 mM Na_3_VO_4_, 1 mM PMSF, 10 µg/ml aprotinin, 10 µg/ml leupeptin and 50 mM NaF. Protein concentration of lysates was measured using a Bradford assay (Bio-Rad). Samples were prepared for Western blotting by boiling in 4X Laemmli sample buffer for 5 min at 95°C. Denatured and normalised samples were resolved by SDS–PAGE running at 25 mA per gel, transferred to methanol-activated Immobilon P PVDF membrane at 300 mA for 90 min using the Mini Trans-Blot Cell wet blotting system. Membranes were blocked in 5% (w/v) powdered milk/TBS-Tween 20 or 5% (w/v) BSA/TBST for 30 min and probed with specific primary antibodies (see below) at 4°C overnight with gentle agitation. Membranes were washed three times in TBST for 5 min and probed with appropriate HRP-conjugated secondary antibody in 5% (w/v) powdered milk/TBST for 1 h at RT. Following another three TBST washes, antibody-antigen complexes were detected by the enhanced chemiluminescence (ECL) system coupled with an autoradiographic film developer. Antibodies used were as follows: Anti-N-IKKα (CST #2682), anti-C-IKKα (Abcam #ab109749), anti-N-IKKβ (Abcam #ab124957), anti-C-IKKβ (CST 2684), anti-p-IKKα/β (S176/180) (CST 2697), anti-IκBα (CST #9242), anti-p-IκBα (S32/36) (CST #9246), anti-p65 (CST #8242), anti-p-p65 (S468) (CST #3039), anti p-p65 (S536) (CST #3033), anti-JNK1 (Santa-Cruz sc-819), anti-p-JNK1/2 (CST #9255), anti-p38 (in-house), anti-p-p38 (T180/Y182) (CST #9211), anti-PARP (CST #9542), anti-FLAG (Sigma–Aldrich F-3165), anti-c-Rel (CST #4727), anti-β-actin (Sigma A5441), anti- β -tubulin (Abcam ab6046), anti-Lamin A/C (Santa-Cruz sc-7292), anti-HSP90 (CST #4874).

### Subcellular fractionation

Cells were harvested by gently scraping in PBS-EDTA (10 mM). Cells were then pelleted and washed with PBS. The washed pellet was then gently resuspended in five pellet volumes of ice-cold Buffer A (10 mM Hepes pH 7.9, 1.5 mM MgCl_2_, 10 mM KCl, 0.1 mM EDTA), supplemented with 1 mM DDT and protease (complete, mini, EDTA-free protease inhibitor cocktail Roche) and phosphatase (PhosSTOP, Roche) inhibitors. Cells were incubated on ice for 15 min then 0.1% (v/v) NP-40 added to release the cytoplasmic contents. A 1 ml dounce homogeniser was used to ensure complete cell lysis. Nuclei were subsequently pelleted at 800 g for 10 min at 4°C and the cytoplasmic fraction (supernatant) transferred to a fresh tube. This supernatant was centrifuged again at 10 000***g*** for 5 min to pellet any contaminating nuclei. NaCl and glycerol were added to the cytoplasmic fraction to final concentrations of 137 mM and 10% (v/v), respectively. The nuclei pellet was washed at least three times in ice-cold Buffer A, prior to addition of 0.7 pellet volumes of RIPA buffer (Sigma), supplemented with >250 U benzonase nuclease (Sigma–Aldrich). Samples were incubated at 4°C for 4 h with end-over-end inversion, with occasional vortexing. The nuclear fraction was subsequently cleared by centrifugation.

### Analysis of cells with sub-G1 DNA content by flow cytometry

Following harvest, cells were pelleted by centrifugation for 5 min (1500 rpm, 4°C), washed in PBS and resuspended in 0.25 ml PBS containing 25 µg RNase and 12.5 µg propidium iodide (PI). Following 30 min incubation at 37°C, samples were passed through a 25-gauge needle to reduce cell clumping. Cell cycle profiles were acquired with an LSRII flow cytometer (BD), using a 488 nm excitation laser line and a 670 nm long pass emission filter to measure binding of PI to DNA, and counting 10 000 cells per sample. Data was analysed using FlowJo v10.

### NF-кB luciferase assays

In 96-well tissue culture plates with opaque sides (Thistle Scientific), HCT116 or SW620 cells were transfected with 0.1 µg/well pGL4.32[luc2P/NF-кB-RE] luciferase experimental-reporter plasmid DNA and 0.01 µg/well constitutively expressed pCMV renilla plasmid DNA (used for normalisation). The following day the relevant treatments were performed. Luciferase activity was measured using the Dual-Luciferase Reporter Assay System (Promega), and on a MicroLumatPlus LB96V reader, according to manufacturer's instructions.

### High-content confocal image acquisition and analysis

Cells were seeded in triplicate in CellCarrier 96-well plates (PerkinElmer) for 48 h prior to fixation with 4% v/v paraformaldehyde/PBS for 15 min at RT. Following three washes in PBS, cells were permeabilised with 0.1% v/v Triton X-100/PBS for 10 min. Cells were washed once in PBS and blocked for 1 h in blocking buffer (1× PBS, 5% v/v goat serum, 0.2% Tween 20, 0.02% w/v sodium azide) at RT. Cells were incubated with primary antibody diluted in antibody dilution buffer at 4°C overnight. Cells were washed three times in PBS and then incubated with secondary antibody diluted in blocking buffer for 1 h at RT in the dark. Cells were washed a final three times in PBS. High-content image acquisition was performed using an In Cell Analyzer 6000 (GE Healthcare). Confocal images were analysed using the CellProfiler software package.

### Inhibitor compounds and cell culture treatments

Initial stocks of BIX02514 were kindly supplied by Prof. Sir Philip Cohen (University of Dundee) and subsequently purchased from Tocris). Stock solutions of drugs were diluted in cell culture medium as appropriate to yield the final drug concentration. All treatments were adjusted to contain the same volume of vehicle. Vehicle-only containing medium was used as a control. Treated cells were incubated at 37°C, 5% CO_2_/95% air for the length of time indicated in the figure legend.

### Statistical analysis

Results, unless otherwise stated, are presented as a mean ± standard deviation (SD) and originate from three independent experiments. Statistical significance was determined by two-way ANOVA (repeated measures) with Tukey or Dunnett post-hoc test using GraphPad 7 where stated. Significance values were given in the figures and set at *P* > 0.05 (ns) and *P* ≦ 0.0001 (***).

## Results

### Generation of IKKα KO, IKKβ KO and IKKα/β DKO HCT116 cells

**Figure 1.**
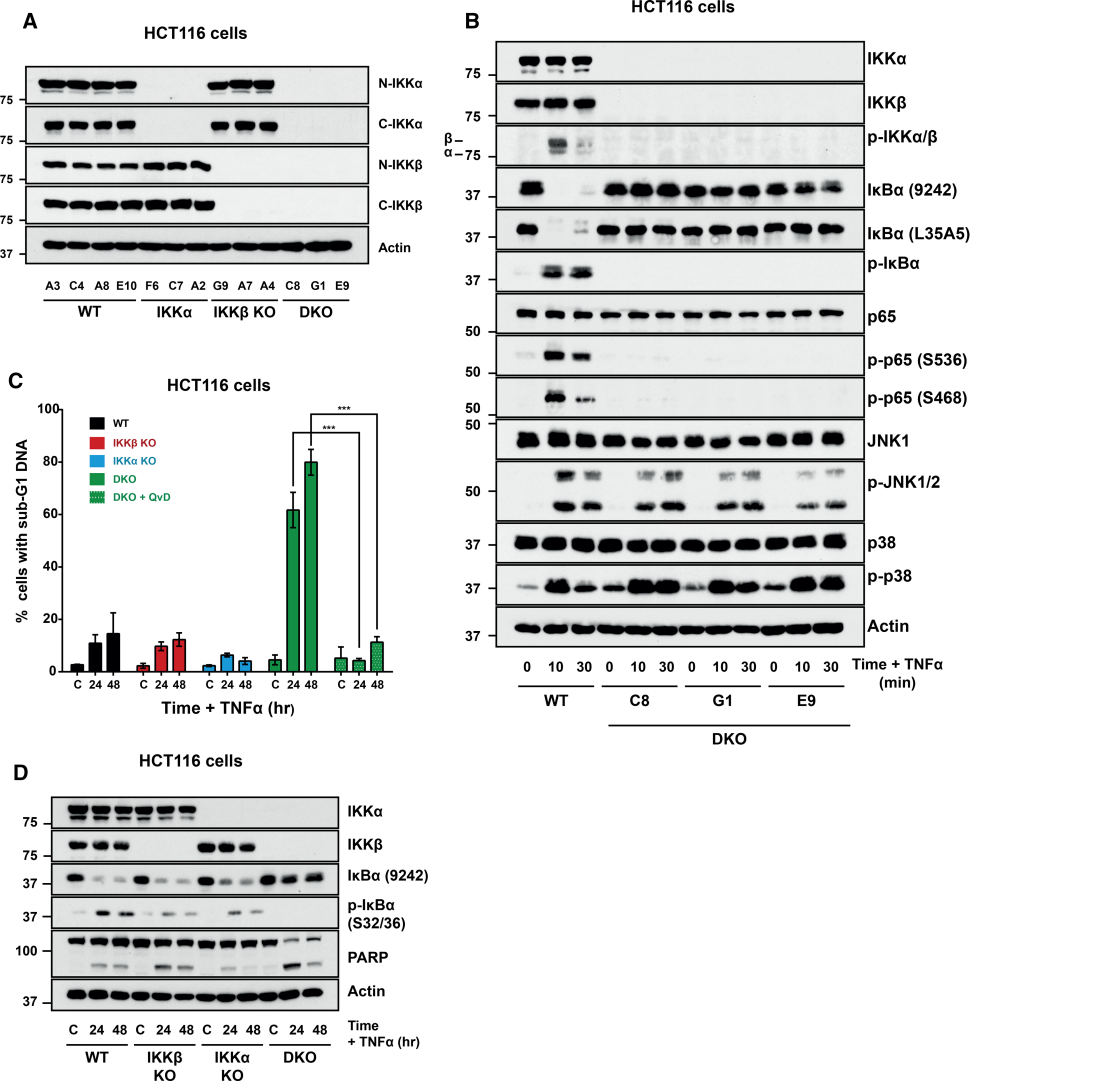
Characterisation of CRISPR–Cas9 IKK knockout HCT116 cells. (**A**) Best candidate clones were expanded and whole-cell lysates generated for independent, single-cell derived WT, IKKα KO, IKKβ KO and IKKα/β DKO CRISPR clones. Lysates were fractioned by SDS–PAGE and Western blotted with the indicated antibodies to confirm absence of expressed protein corresponding to targeted gene. Three clones each of IKKα KO (F6, C7, A2), IKKβ KO (G9, A7, A4) and IKKα/β DKO (C8, G1, E9) were selected for validation and characterisation. In parallel, we derived four wild-type clones (A3, C4, A8, E10) that had been transfected with Cas9 but no guide. Data are from a single experiment representative of three independent Western blots giving similar results. (**B**) WT clone (A3) and three independent IKKα/β DKO clones were seeded in their normal growth medium for 48 h, prior to treatment with 10 ng/ml recombinant TNFα for the indicated timepoints. Whole-cell extracts were prepared, fractionated by SDS–PAGE and Western blotted with the indicated antibodies. Data are from a single experiment. p, phospho-. (**C**) WT (A3), IKKα KO (F6), IKKβ KO (G9) and IKKα/β DKO (C8) HCT116 cells were seeded in triplicate in normal growth medium for 24 h prior to treatment with 10 ng/ml TNFα for the indicated timepoints (24 and 48 h). Caspase-dependent cell death was blocked by pre-treating cells for 30 min with 10 µM of the pan-caspase inhibitor, QVD-OPh. Cells that were sub confluent at the point of harvest, were fixed, stained with propidium iodide and percentage sub-G1 population (as a crude marker of cell death) assessed by flow cytometry. Results are mean ± SD of the three independent experiments. Significance testing was performed using two-way ANOVA (repeated measures) with Tukey post-hoc testing. *P *< 0.0001 (***). (**D**) The same clones were treated as in (**C**) and whole lysates prepared and Western blotted with the indicated antibodies. Data are from a single experiment representative of two showing similar results.

HCT116 cells are an epithelial colorectal cancer cell line established in 1981 from an adult male patient. HCT116 cells are wild type for TP53 but harbour a KRAS^G13D^ and PIK3CA^H1047R^ mutation as well as inactivating mutations in CTNNB1, CDKN2A/p14ARF, MLH1. IKKα or IKKβ knockout (KO) and IKKα/β double knockout (DKO) HCT116 cell lines were generated by CRISPR–Cas9 gene editing and single-cell cloning (see Materials and Methods). Clones A3, C4, A8 and E10 were confirmed as WT. Clones F6 and C7, derived from cells expressing gRNA 24 and clone A2, derived from cells expressing gRNA 25, were confirmed as IKKα KO. Clones G9 and A7, derived from cells expressing gRNA 87 and clone A4, derived from cells expressing gRNA 76, were confirmed as IKKβ KO. Clones C8 and G1, derived from cells expressing both gRNA 25 and gRNA 87, and clone E9, derived from cells expressing both gRNA 24 and gRNA 87, were confirmed as IKKα/β DKO. Sanger sequencing confirmed the presence of homozygous frameshift mutations and likely premature stop codons (Supplementary Figures S1 and S2). Immunoblotting with N-terminal or C-terminal antibodies against IKKα or IKKβ confirmed that each KO clone was null for full-length protein (Figure 1A and Supplementary Figure S3); no compensatory up-regulation of the remaining IKK subunit was observed in single KO clones. IKKα KO and DKO clones exhibited a greater than 2-fold reduction in *IKKα* mRNA; a similar reduction in *IKKβ* mRNA was seen in the IKKβ KO and DKO clones (Supplementary Figure S4A,B).

To assess the consequences of IKK knockout we examined the signalling response to TNFα, focusing first on the IKKα/β DKO cells. All three IKKα/β DKO clones lacked TNFα-inducible IKK activity, judged by phosphorylation of known substrates IκBα and p65 (Figure 1B). The absence of p65 phosphorylation at serine 536 (S536) and S468 in DKO clones indicated that in the absence of IKKα and IKKβ these sites cannot be phosphorylated in response to TNFα by other kinases, such as TBK1 and IKKε [37]. The IKKα/β DKO clones also exhibited a complete loss of TNFα-inducible NF-κB transcriptional activity (see below). IKKα/β DKO clones exhibited normal phosphorylation of JNK at T183/Y185 in response to TNFα. However, the DKO clones exhibited increased basal p38 phosphorylation at T180/Y182, and TNFα-stimulated p38 phosphorylation was more persistent (Figure 1B).

Activation of NF-κB is cytoprotective during TNFα stimulation [38,39]. Consistent with this, IKKα/β DKO cells exhibited a striking caspase-dependent cell death following TNFα stimulation (Figure 1C) that was accompanied by caspase activation and PARP cleavage (Figure 1D), consistent with cell death by apoptosis. Thus, as expected, IKKα/β DKO cells were completely defective for TNFα-dependent activation of IKK and were sensitised to TNFα-driven apoptosis.

### A prominent role for IKKα in canonical NF-κB signalling downstream of TNFα

**Figure 2.**
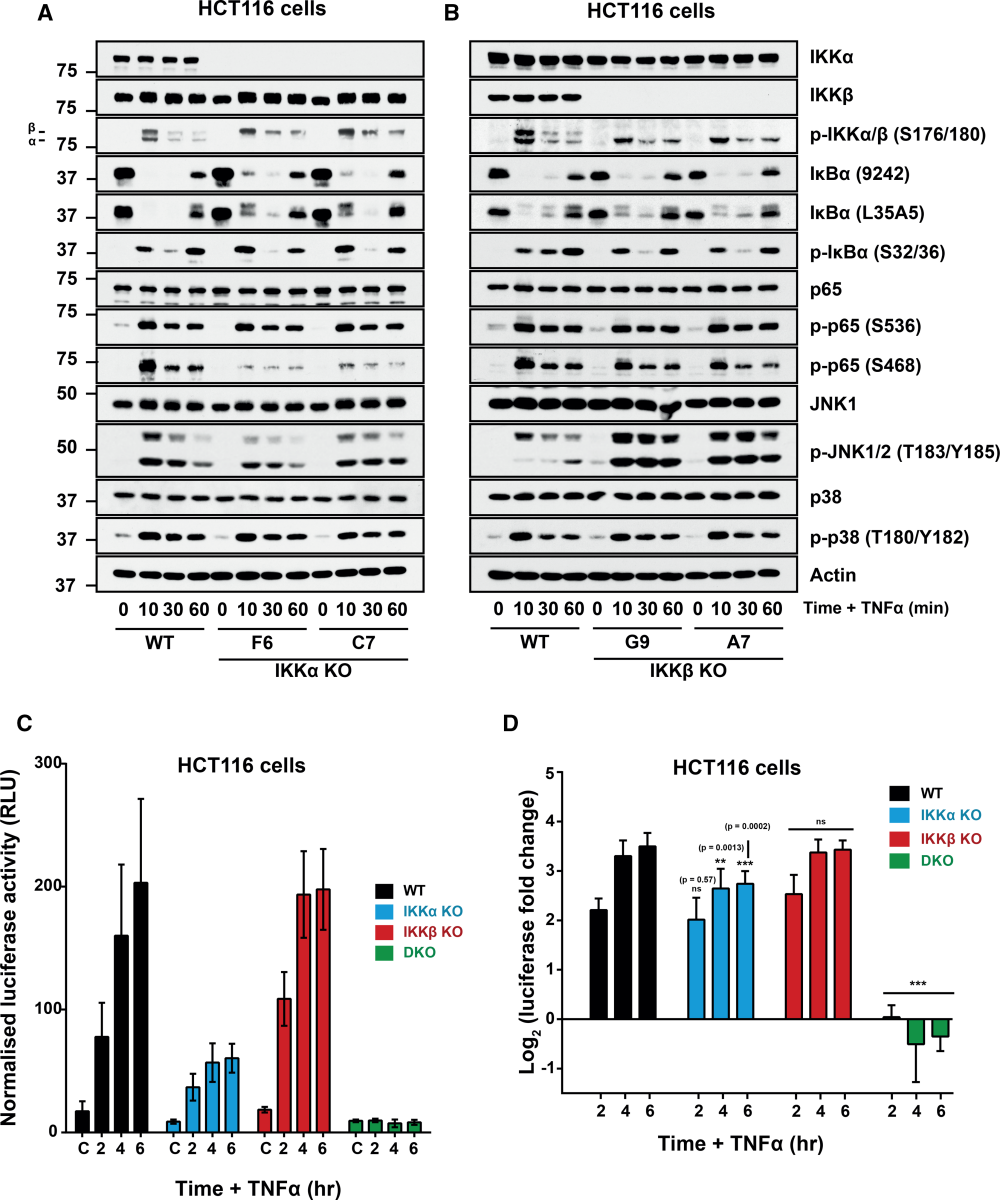
Redundant and non-redundant functions for IKKα and IKKβ in NF-κB activation. (**A**) WT clone (A3) and two independent IKKα KO clones or (**B**) WT clone (A3) and two independent IKKβ KO clones were seeded in their normal growth medium for 48 h, prior to treatment with 10 ng/ml recombinant TNFα for the indicated timepoints. Whole-cell extracts were prepared, fractionated by SDS–PAGE and Western blotted with the indicated antibodies. Similar results were seen for the two other WT clones (A8 and E10) and the other IKKα (A2) and IKKβ (A4) KO clones (data not shown). (**C** and **D**) WT, IKKα KO, IKKβ KO and IKKα/β DKO HCT116 cells were seeded in antibiotic-free growth medium overnight prior to transient transfection with NF-κB-RE firefly luciferase and renilla luciferase plasmids. The following day, cells were treated with 10 ng/ml recombinant TNFα for the indicated time periods. Firefly luciferase was normalised to renilla luciferase and data expressed as log_2_(fold change in TNFα-induced luciferase activity relative to the relevant, matched untreated condition). Results are mean ± SD of three independent experiments, each of which was performed with three independent clones, seeded in triplicate (WT — A3, A8 and E10. IKKα KO — F6, C7, A2. IKKβ KO — G9, A7, A4 and IKKα/β DKO — C8, G1, E9). Significance testing was performed using two-way ANOVA (repeated measures) with Tukey post-hoc test. *P* values relate to comparison with corresponding timepoint(s) of WT control. *P* < 0.0001 (***).

We next examined TNFα responses in IKKα or IKKβ KO cells, comparing with WT cells (Figure 2A,B). IKKα and IKKβ were phosphorylated on their activation loops after 10 minutes of TNFα treatment; the antibody used (p-S176/S180) detects IKKα only when phosphorylated at S176/180 and IKKβ only when phosphorylated at S177/181. The KO clones highlighted the specificity of this antibody; the upper band being IKKβ (S177/181) and the lower band IKKα (S176/180) (Figure 2A,B). Interestingly, IKKβ S177/181 phosphorylation was enhanced in IKKα KO cells relative to WT whilst IKKα S176/180 phosphorylation was also enhanced in the IKKβ KO clones relative to WT cells. This was also seen over longer time courses, where IKKβ S177/181 and IKKα S176/180 phosphorylation was both enhanced and more sustained in the IKKα KO and IKKβ KO cells, respectively (Supplementary Figure S5A,B). In WT cells IκBα was phosphorylated at S32/36 and degraded within 10 min of TNFα treatment and IκBα protein levels were restored by 60 min, reflecting the NF-κB-driven negative feedback loop [40]. Both the IKKα KO and IKKβ KO cells exhibited a similar, modest delay in IκBα degradation relative to WT cells after 10 and 30 min TNFα treatment, but IκBα levels were restored after 60 min in both IKKα KO and IKKβ KO cells. These results indicate that IKKα and IKKβ are functionally redundant for IκBα phosphorylation in HCT116 cells, each compensating for the other.

p65 NF-κB was rapidly phosphorylated at S468 and S536 following TNFα treatment; phosphorylation of S536 was unaffected by IKKα KO or IKKβ KO, suggesting that IKKα and IKKβ are fully redundant for phosphorylating S536, consistent with both being able to phosphorylate S536 [5,41,42]. Interestingly, basal phosphorylation of S536 was absent from IKKα KO clones but was unaffected in IKKβ KO clones, suggesting that IKKα might contribute to basal phosphorylation of S536. Notably, TNFα-driven p65 S468 phosphorylation was strongly diminished in IKKα KO clones, but largely unaffected by IKKβ KO, suggesting that IKKα, not IKKβ, is critical for phosphorylation at this site in response to TNFα. This is in direct contrast with the literature where IKKβ has been suggested to be the dominant kinase phosphorylating S468 in response to TNFα and IL-1, with little if any contribution from IKKα [43]. To examine the effect of IKK KO on NF-κB transcriptional activity cells were transfected with a tandem NF-κB-dependent luciferase reporter (NF-κB:Luc) (Figure 2C,D). Both basal and especially TNFα-induced NF-κB activity was substantially reduced in IKKα KO clones relative to WT (Figure 2C,D) but unaffected by IKKβ KO. IKKα/β DKO clones were completely defective for TNFα-induced NF-κB activity (Figure 2C,D), consistent with the complete absence of IKK activity (Figure 1D).

Phosphorylation of p38 was unaffected by knockout of either IKKα or IKKβ (Figure 2A,B), suggesting that the enhanced p38 phosphorylation observed in DKO clones (Figure 1B) reflected the complete loss of IKK kinase activity and/or NF-κB activity. Activating phosphorylation of JNK1/2 in response to TNFα was unaffected by IKKα KO but greatly enhanced by IKKβ KO.

### siRNA or selective IKKβ inhibition in IKK KO cells confirms a prominent role for IKKα in canonical NF-κB signalling

**Figure 3.**
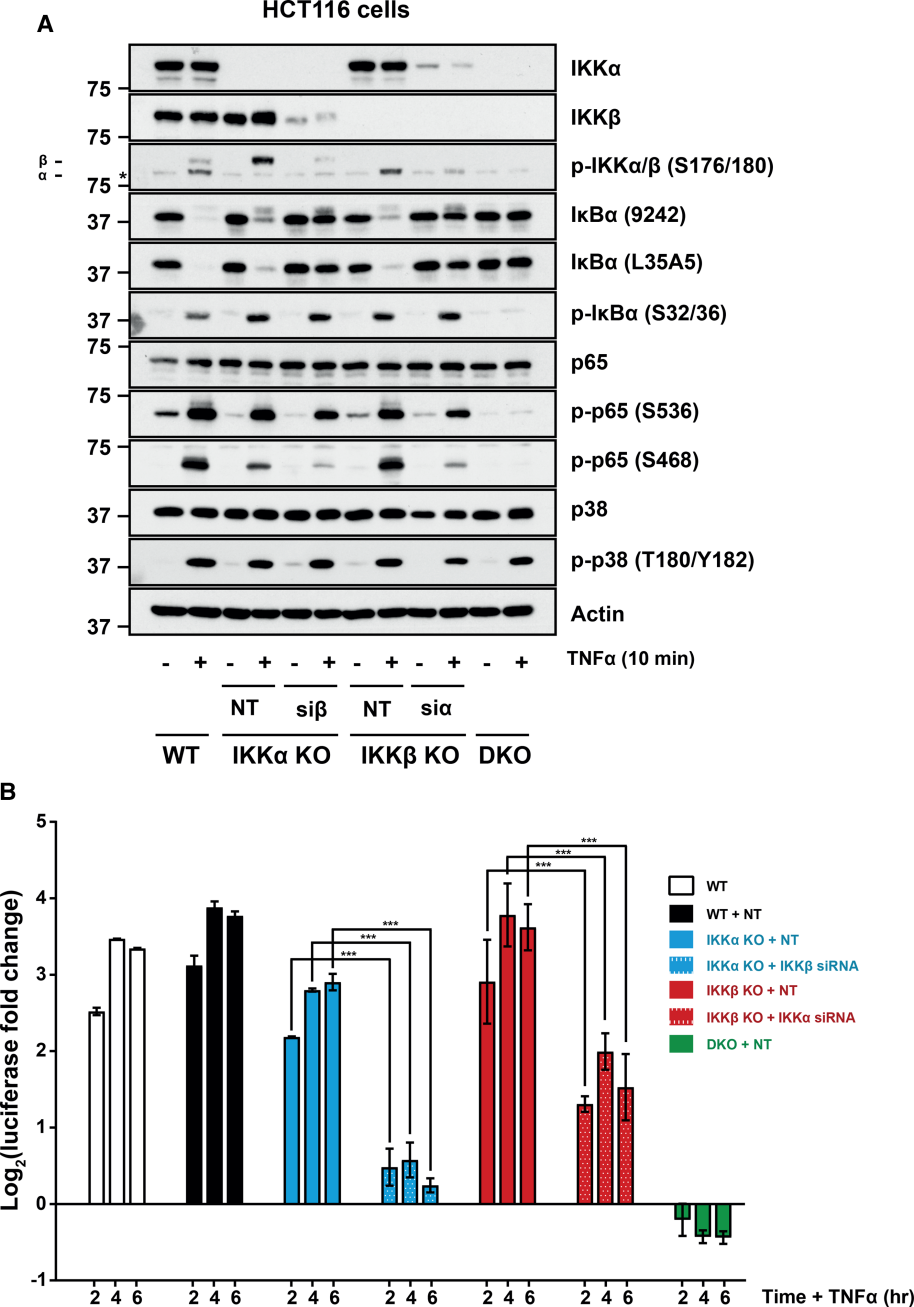
Knocking down the remaining IKK subunit or IKKβ inhibition in single KO clones confirms redundant functions of IKKs. (**A**) WT (A3), IKKα KO (F6), IKKβ KO (G9) and IKKα/β DKO (C8) HCT116 cells were transfected with non-targeting (NT) siRNA, IKKα-specific siRNA (siα) or IKKβ-specific siRNA (siβ). Forty-eight hours later, cells were treated with 10 ng/ml TNFα for 10 min and whole-cell lysates were prepared and Western blotted with the indicated antibodies. Data are from a single experiment representative of two giving similar results. *Faint non-specific band running at similar molecular weight to P-IKKα. (**B**) WT, IKKα KO, IKKβ KO and IKKα/β DKO HCT116 cells were transfected as above. Twenty-four hours later, cells were transiently transfected with NF-κB-RE firefly luciferase and renilla luciferase plasmids. The following day, cells were treated with 10 ng/ml TNFα for 2, 4 or 6 h and firefly luciferase normalised to renilla luciferase luminescence and expressed as log_2_(fold change in TNFα-induced luciferase activity relative to the relevant, matched untreated condition). Results are mean ± SD of two independent experiments using the same KO clones as in (**A**), each performed in technical triplicate. Significance testing was performed using two-way ANOVA (repeated measures) with Tukey post-hoc test. *P* < 0.0001 (***).

The preceding results suggested a prominent role for IKKα in canonical NF-κB activation. To confirm these results by orthogonal approaches IKKα KO or IKKβ KO clones were treated with siRNA targeting the remaining IKK, knocking down IKKβ in IKKα KO cells or vice versa (Figure 3A,B). This was sufficient to almost completely block the degradation of IκBα after 10 min TNFα treatment in the IKKα or IKKβ KO clones (Figure 3A); thus, the residual IKK activity observed in these KO clones was due to the remaining IKK kinase confirming that IKKα and IKKβ are functionally redundant in TNFα dependent degradation of IκBα. Phosphorylation of p65 at S536 and S468 was also diminished following the knockdown of the remaining IKK subunit in both the IKKα and IKKβ KO clones but IKKα KO or IKKα knockdown had the strongest effect on S468 phosphorylation. NF-κB:Luc assays also demonstrated that knocking down the remaining IKK subunit caused a significant reduction in TNFα-induced NF-κB activity (Figure 3B).

**Figure 4.**
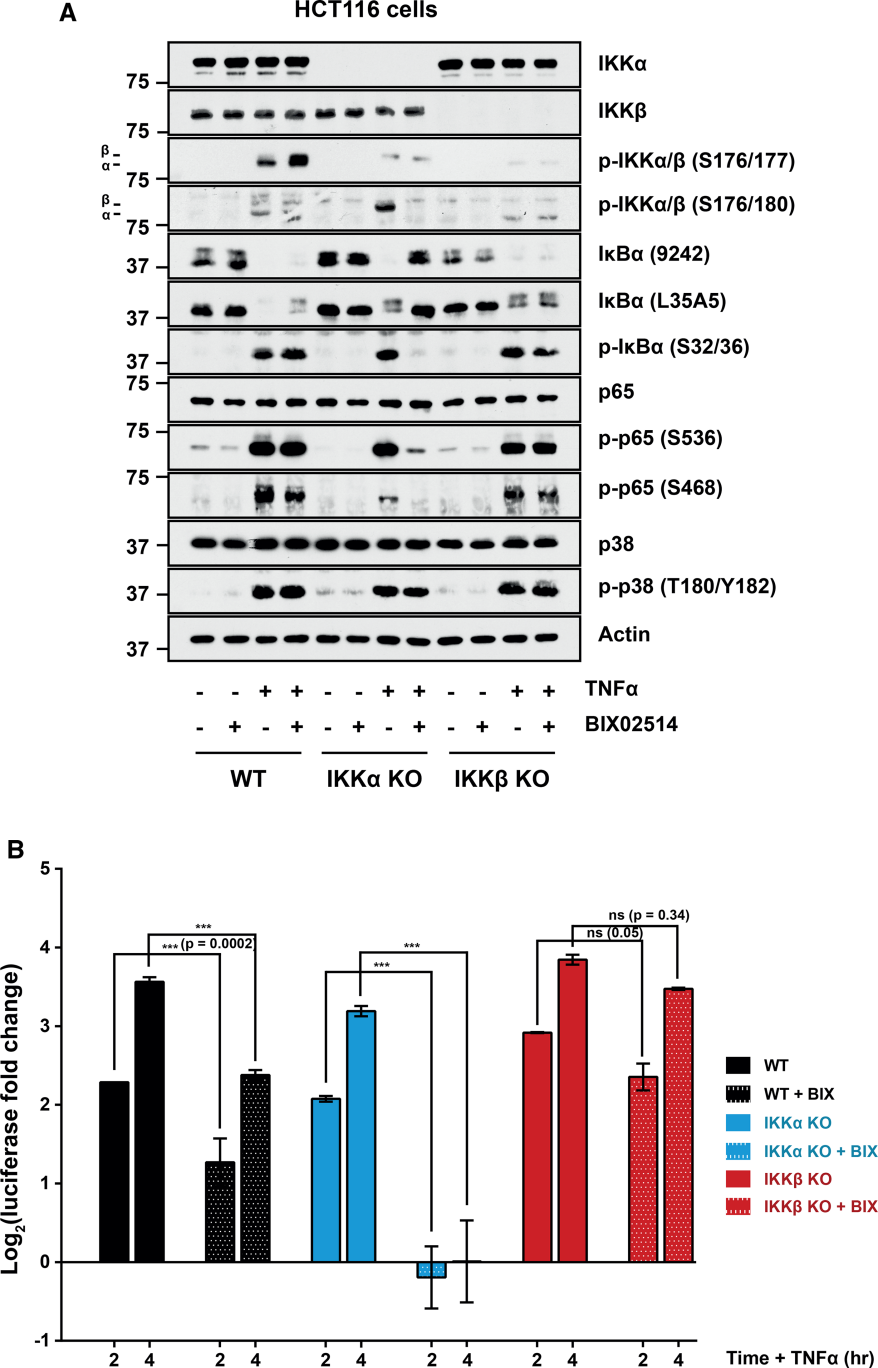
Selective IKKβ inhibition confirms a prominent role for IKKα in canonical NF-κB signalling. (**A**) WT, IKKα KO and IKKβ KO HCT116 cells were seeded for 48 h prior to treatment with 10 µM BIX02514 or DMSO vehicle control for 30 min. Cells were then treated with TNFα (10 min) and processed for western blot. (**B**) WT, IKKα KO and IKKβ KO HCT116 cells were transiently transfected with reporter plasmids. The following day, cells were treated with BIX02514 or DMSO vehicle control for 30 min, prior to treatment with TNFα for 2 or 4 h. Firefly luciferase luminescence was normalised as above and expressed as log_2_(fold change in TNFα-induced luciferase activity relative to the relevant, matched untreated condition). Results are mean ± SD of two independent experiments using the same KO clones as in (**A**) each performed in technical triplicate. Significance testing was performed using two-way ANOVA (repeated measures) with Tukey post-hoc test. *P* < 0.0001 (***). BIX, BIX02514.

To complement this siRNA approach, we used the IKKβ inhibitor BIX02514, which is ∼300-fold selective for IKKβ (*in vitro* IC_50_ of 300 nM) versus IKKα (*in vitro* IC_50_ of 100 µM) (reviewed in 23). BIX02514 had only a modest effect on TNFα-induced degradation of IκBα in WT cells but this resulted in a significant 2-fold decrease in the TNFα-induced NF-κB activity suggesting that IKKα and IKKβ both contribute to induction of NF-κB in wild-type cells (Figure 4A,B). BIX02514 had no effect on the phosphorylation of p65 at S536 or S468 in WT cells, suggesting that IKKα phosphorylates these sites in response to TNFα. In IKKα KO cells BIX02514 completely inhibited IκBα phosphorylation, IκBα degradation, phosphorylation of p65 at S536, the residual phosphorylation of S468 and TNFα-induced NF-κB:Luc activity (Figure 4A,B). These results strongly suggest that the IKK activity observed in IKKα KO cells is IKKβ and that both kinases normally contribute to TNFα-stimulated NF-κB activation. BIX02514 had no effect on TNFα-induced phosphorylation and degradation of IκBα, or the phosphorylation of p65 in IKKβ KO cells, confirming that IKK activity in these cells is not due to residual IKKβ activity but rather due to IKKα. Indeed, BIX02514 had no significant effect on the induction of NF-κB:Luc in IKKβ KO cells, again indicating that IKKα could fully compensate for IKKβ loss (Figure 4B).

TNFα-driven NF-κB transcriptional activity could be restored to IKKα/β DKO cells by transiently re-expressing WT IKKα or WT IKKβ constructs but not their kinase dead (KD) counterparts (Supplementary Figure S6A). Notably IKKα was more effective at restoring NF-κB:Luc than IKKβ, despite IKKβ being expressed at slightly higher levels (Supplementary Figure S6A,B); re-expression of both WT IKKα and IKKβ had an additive effect. WT IKKα re-expression in IKKα KO cells rescued TNFα-induced NF-κB activation, whereas overexpression of WT IKKβ had no effect (Supplementary Figure S6B,C). This further highlights the importance of IKKα in the canonical NF-κB response to TNFα in these cells. KD IKKβ strongly inhibited TNFα-induced NF-κB activation in WT cells and IKKβ KO cells, and inhibited the restored TNFα-inducible NF-κB activation in DKO cells when co-expressed with WT IKKα. KD IKKβ also inhibited the induction of NF-κB in IKKα KO cells, indicating that KD IKKβ acts in a dominant negative fashion towards both WT IKKβ and IKKα consistent with previous studies [28]. KD IKKα also inhibited the induction of NF-κB in WT cells, albeit not to the same extent as KD IKKβ, and also inhibited the induction of NF-κB in IKKα and IKKβ KO cells, indicating that kinase inactive IKKα also acts as a dominant negative to inhibit both IKKα and IKKβ.

### IKKα KO cells exhibit a far greater defect in TNFα-induced p65 nuclear translocation than IKKβ KO cells

**Figure 5.**
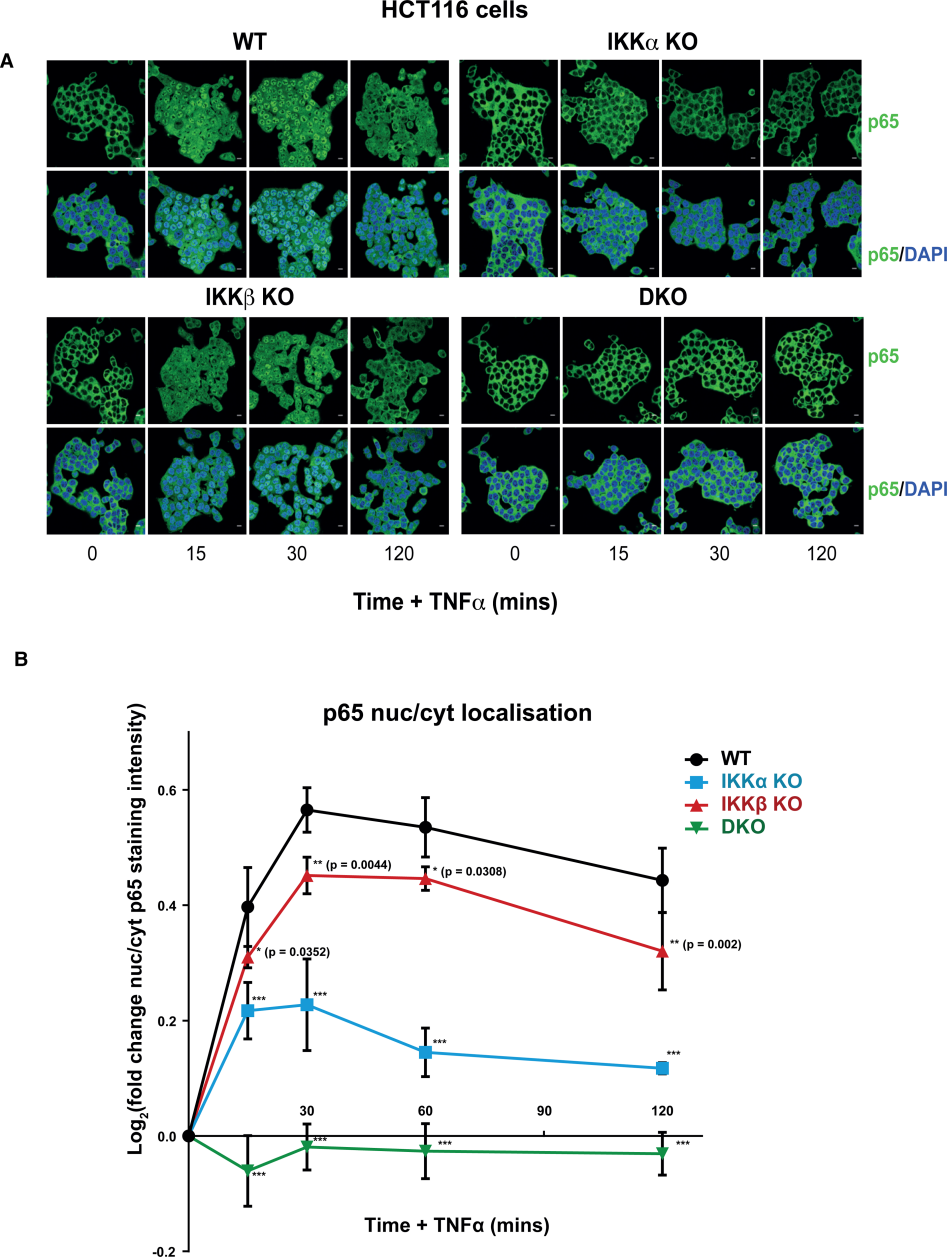
A critical role for IKKα in TNFα-induced p65 nuclear translocation. WT, IKKα KO, IKKβ KO and IKKα/β DKO HCT116 cells were seeded in normal growth medium for 48 h, prior to treatment with 10 ng/ml TNFα for the indicated times. Immunofluorescence staining of cells was performed with anti-p65 antibody (green) and nuclei were stained with DAPI (blue). (**A**) Representative images are shown for time zero, 15, 30 and 120 min of TNFα stimulation. (**B**) Cells were seeded in 96 well plates in normal growth medium for 48 h, prior to treatment with 10 ng/ml TNFα for the indicated timepoints. Cells were stained with anti-p65 and DAPI and confocal images captured using the In Cell Analyzer 6000 high-content imaging system (20× objective). p65 staining intensity analysis was performed using CellProfiler software. Data is presented as the log_2_ transformed fold change in nuclear:cytoplasmic p65 staining intensity. Data are mean ± SD of three independent CRISPR–Cas9 KO clones in each case. Significance testing relative to WT control cells performed using two-way ANOVA (repeated measures) with Dunnett post-hoc test. *P* < 0.0001 (***). Scale bar, 10 µm.

**Figure 6.**
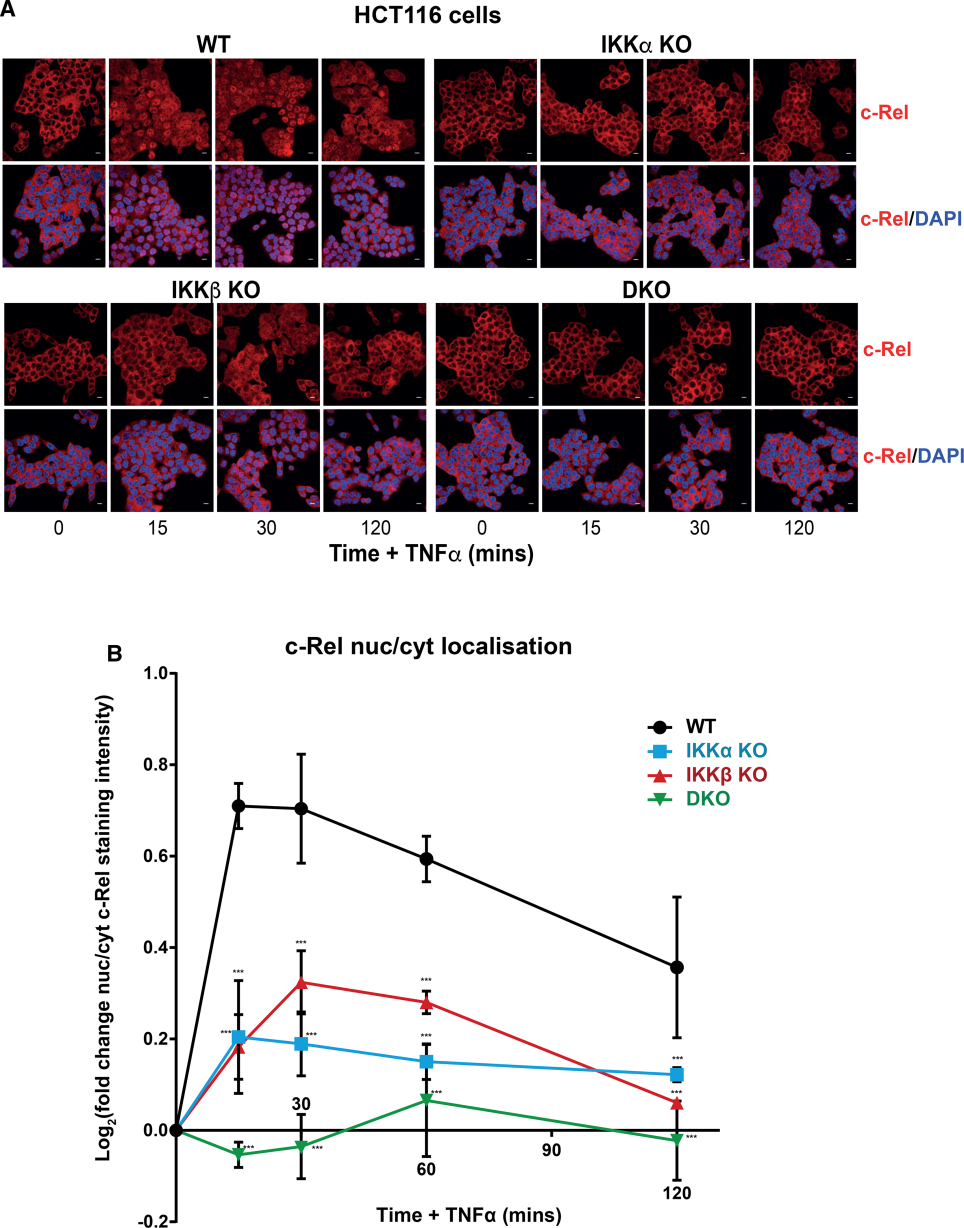
Redundant roles for IKKα and IKKβ in TNFα-induced c-Rel nuclear translocation. WT, IKKα KO, IKKβ KO and IKKα/β DKO HCT116 cells were seeded in normal growth medium for 48 h, prior to treatment with 10 ng/ml TNFα for 15, 30 and 120 min. Immunofluorescence staining of cells was performed with anti-c-Rel (red) and nuclei were stained with DAPI (blue). (**A**) Representative images are shown for time zero, 15, 30 and 120 min of TNFα stimulation. (**B**) Cells were seeded in 96 well plates in normal growth medium for 48 h, prior to treatment with 10 ng/ml TNFα for the indicated timepoints. Cells were stained with anti-c-Rel and DAPI and confocal images captured using the In Cell Analyzer 6000 high-content imaging system (20× objective). c-Rel staining intensity analysis was performed using CellProfiler software. Data is presented as the log_2_ transformed fold change in nuclear:cytoplasmic c-Rel staining intensity. Data are mean ± SD of three independent CRISPR–Cas9 KO clones in each case. Significance testing relative to WT control cells performed using two-way ANOVA (repeated measures) with Dunnett post-hoc test. *P* < 0.0001 (***). Scale bar, 10 µm.

IKKα and IKKβ were redundant for IκBα phosphorylation and degradation (Figure 2A,B) but only IKKα KO cells exhibited a significant reduction in TNFα-induced NF-κB:Luc. Consequently, we examined the nuclear translocation of NF-κB subunits by immunostaining for p65 (Figure 5A,B). In WT HCT116 cells TNFα stimulated p65 accumulation within the nucleus, with a peak after 30 min, followed by a progressive decline. Consistent with the NF-κB:Luc results, IKKα KO cells exhibited a striking reduction in p65 nuclear localisation at all time points examined whereas IKKβ KO cells exhibited a similar p65 nuclear localisation response to WT cells; IKKα/β DKO cells exhibited no p65 nuclear localisation in response to TNFα (Figure 5A). These observations were quantified in three independent clones of each of the four genotypes (WT, IKKα KO, IKKβ KO and IKKα/β DKO) through high-content confocal image analysis (Figure 5B) and confirmed that IKKα KO clones exhibited a strong, statistically significant reduction in TNFα-induced nuclear localisation of p65, whereas IKKβ KO clones exhibited a far more modest reduction in nuclear p65. Interestingly, when we analysed another NF-κB subunit, c-Rel, IKKα KO and IKKβ KO clones exhibited very similar defects in TNFα-induced nuclear localisation relative to WT suggesting each IKK made a similar contribution to c-Rel nuclear translocation (Figure 6A,B); IKKα/β DKO cells exhibited no c-Rel nuclear localisation in response to TNFα.

We confirmed this analysis using subcellular fractionation as an orthogonal assay; fractions were well resolved as judged by β-tubulin and Lamin A/C (Supplementary Figure S7A,B). Consistent with the immunofluorescence analysis, TNFα-induced nuclear translocation of p65 was greatly reduced in IKKα KO cells compared with WT whereas p65 translocation was only slightly reduced in IKKβ KO cells. In contrast, IKKα KO and IKKβ KO cells exhibited similar defects in the TNFα-induced nuclear translocation of c-Rel relative to WT cells, again confirming the immunofluorescence results. The greater defect in p65 nuclear translocation in IKKα KO cells relative to IKKβ KO cells was not a consequence of a greater defect in TNFα-induced IκBα degradation as this was comparable between IKKα KO and IKKβ KO cells. Previous studies have reported a significant proportion of full-length IKKα localised to the nucleus of HCT116 cells [44], while others have reported TNFα-inducible nuclear translocation of IKKα [45]. A proportion of full-length IKKα was detected in the nucleus of WT HCT116 cells, but only after over exposure of the blot (Supplementary Figure S7C); this nuclear localisation was confirmed, however, through immunofluorescence staining (Supplementary Figure S7D). However, no increase in nuclear translocation of IKKα after TNFα treatment was observed (Supplementary Figure S7C).

Collectively, these results demonstrate that IKKα KO and IKKβ KO cells are similarly defective in TNFα-induced nuclear translocation of c-Rel, whereas IKKα KO cells exhibit a major defect in p65 nuclear translocation which is not seen in IKKβ KO cells. These subunit-selective differences are not explained by differences in IκBα degradation but are likely to be a major factor accounting for the reduced TNFα-inducible NF-κB transcriptional activation observed in IKKα KO cells.

### Loss of IKKα is more detrimental to IL-1α induced NF-κB signalling than loss of IKKβ

**Figure 7.**
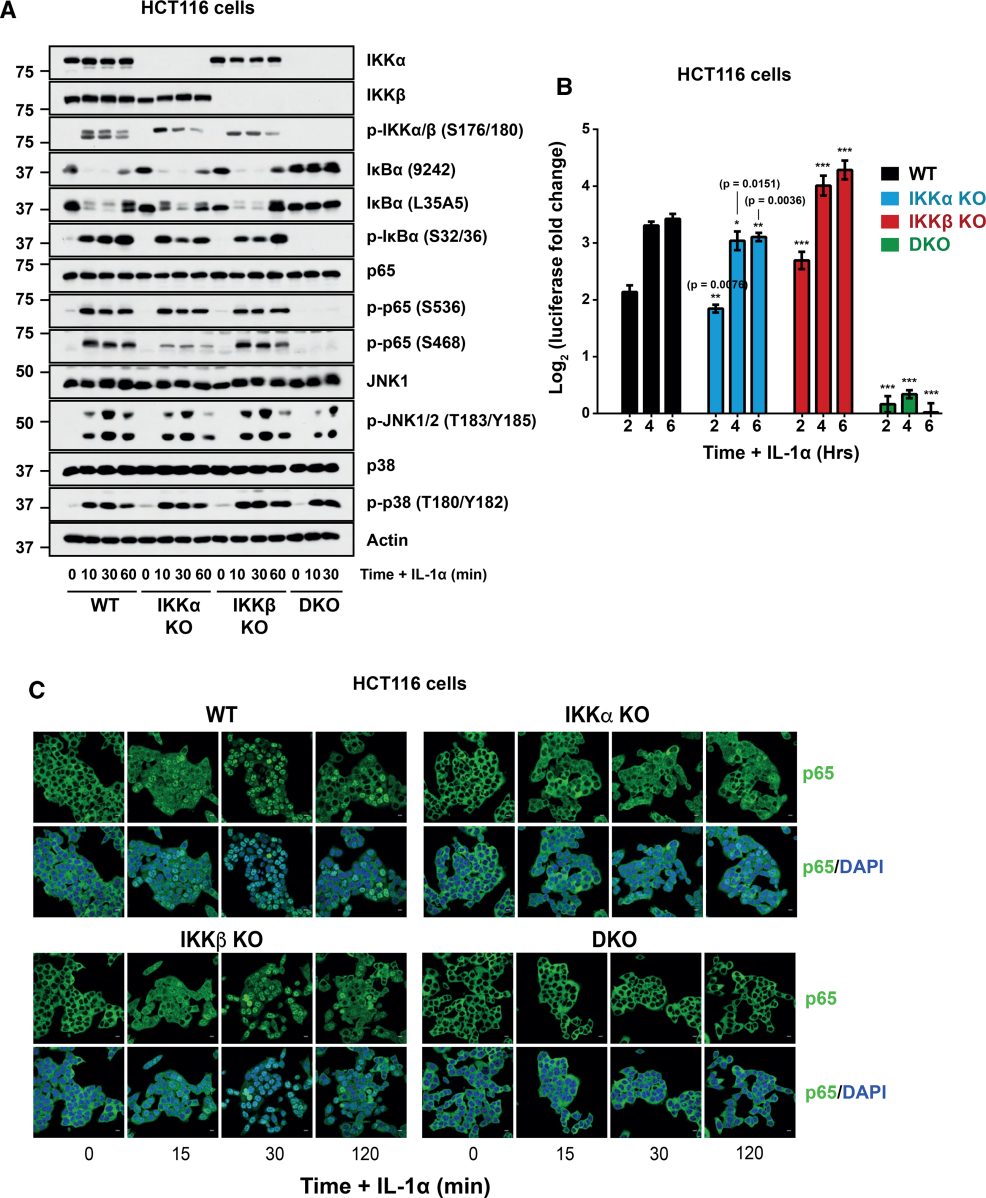
A major role for IKKα in NF-κB signalling in response to IL-1α. (**A**) WT (A3), IKKα KO (F6), IKKβ KO (G9) and IKKα/β DKO (C8) HCT116 cells were seeded in their normal growth medium for 48 h, prior to treatment with 25 ng/ml IL-1α for 10, 30 and 60 min. Whole-cell extracts were prepared, fractionated by SDS–PAGE and Western blotted with the indicated antibodies. (**B**) The same clones as in (**A**) were seeded overnight prior to transient transfection with NF-κB-RE firefly luciferase and renilla luciferase plasmids. The following day, cells were treated with 25 ng/ml IL-1α for 2, 4 and 6 h. Firefly luciferase was normalised to renilla luciferase and data expressed as log_2_(fold change in IL-1α-induced luciferase activity relative to the relevant, matched untreated condition). Results are mean ± SD of three independent experiments, each seeded in technical triplicate. Significance testing was performed using two-way ANOVA (repeated measures) with Tukey post-hoc test. *P* values relate to comparison with corresponding timepoint(s) of WT control. *P* < 0.0001 (***). (**C**) The same clones as in (**A**) were seeded in normal growth medium for 48 h, prior to treatment with 25 ng/ml IL-1α for 15, 30 or 120 min. Immunofluorescence staining of cells was performed with anti-p65 (green) and nuclei with DAPI (blue). Scale bar, 10 µm.

The importance of IKKα or IKKβ in canonical NF-κB signalling can depend on the stimulus [5,26] so we also examined responses to IL-1α (Figure 7A). As with TNFα, both IKKα and IKKβ KO cells, but not IKKα/β DKO, cells responded to IL-1α to activate the canonical NF-κB pathway. The degradation of IκBα in response to IL-1α was similar in IKKα KO and IKKβ KO cells, indicating redundant roles for both kinases. Similar to TNF-α, the IL-1α-induced phosphorylation of p65 at S536 was largely unaffected in IKKα KO or IKKβ KO cells, but basal phosphorylation at this site was again reduced in IKKα KO cells. Furthermore, IL-1α-induced p65 S468 phosphorylation was greatly reduced in IKKα KO cells, while IKKβ KO cells were largely unaffected. Unlike TNFα, however, IKK KO had no effect on the phosphorylation of JNK or p38 in response to IL-1α. In common with the response to TNFα, IKKα KO cells exhibited a significantly lower IL-1α-induced NF-κB:Luc response relative to WT; this was not observed in IKKβ KO cells where the transcriptional response to IL-1α was slightly enhanced relative to WT (Figure 7B). As expected, IKKα/β DKO cells exhibited no response to IL-1α stimulation. Consistent with these results, IKKβ KO cells exhibited no defect in IL-1α induced p65 nuclear translocation relative to WT cells, whereas IKKα KO cells exhibited a strong defect (Figure 7C). Collectively, these results indicate that HCT116 cells are more dependent upon IKKα than IKKβ for nuclear entry of p65 and activation NF-κB:Luc signalling in response to either TNFα or IL-1α.

### A prominent role for IKKα in canonical NF-κB signalling in SW620 cells

**Figure 8.**
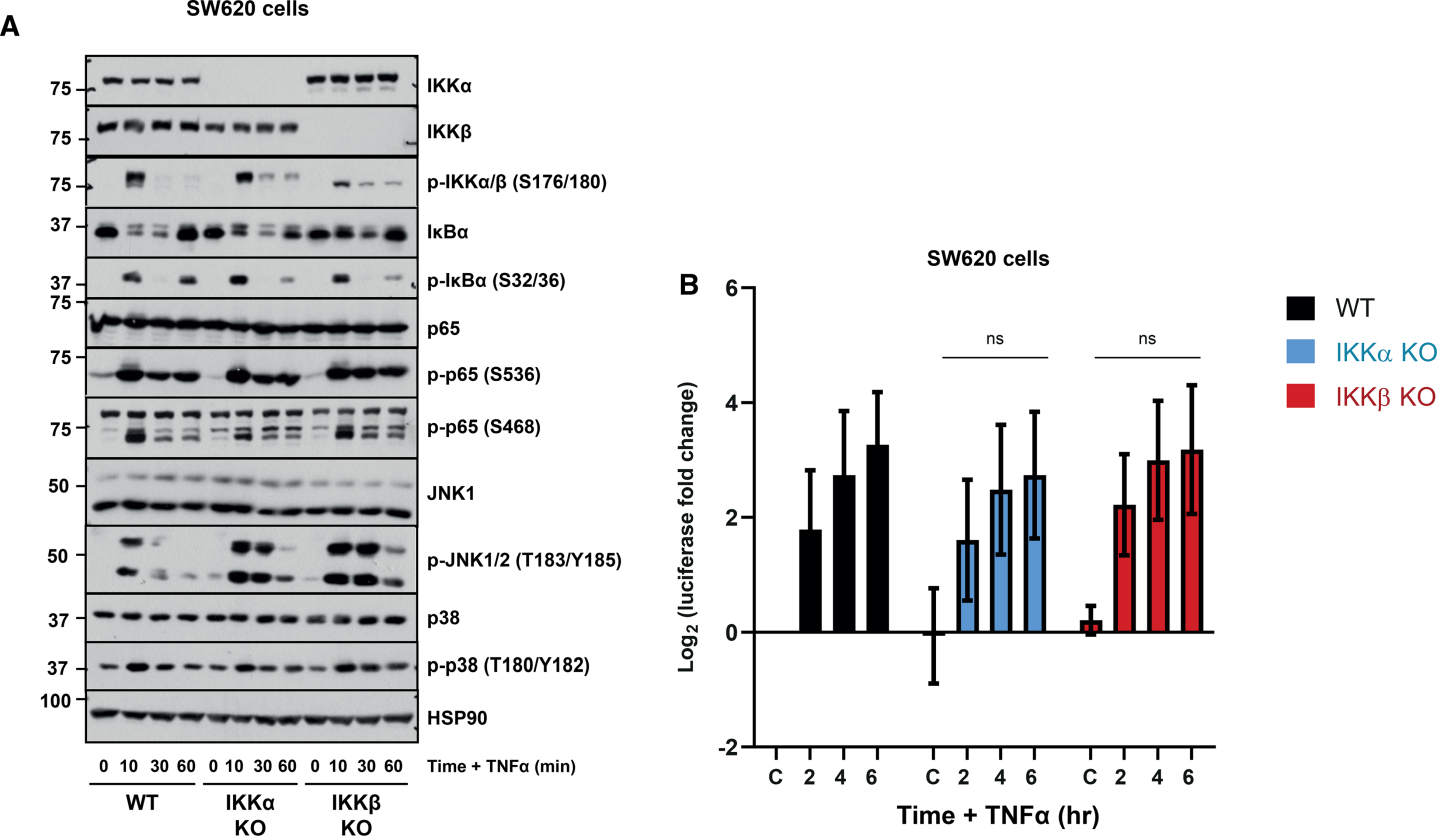
Redundant roles for IKKα and IKKβ in canonical NF-κB signalling in SW620 cells. (**A**) WT, IKKα KO or IKKβ KO SW620 cells were seeded in their normal growth medium for 48 h, prior to treatment with 10 ng/ml TNFα for 10, 30 and 60 min. Whole-cell extracts were prepared, fractionated by SDS–PAGE and Western blotted with the indicated antibodies. (**B**) WT, IKKα KO or IKKβ KO SW620 cells were seeded overnight prior to transient transfection with NF-κB-RE firefly luciferase and renilla luciferase plasmids. The following day, cells were treated with 10 ng/ml TNFα for 2, 4 and 6 h. Firefly luciferase was normalised to renilla luciferase and data expressed as log_2_(fold change in IL-1α-induced luciferase activity relative to the relevant, matched untreated condition). Results are mean ± SD of three independent experiments, each seeded in technical triplicate.

Finally, we used CRISPR–Cas9 gene editing to generate clones lacking IKKα or IKKβ in a second, independent colorectal adenocarcinoma cell line, SW620, isolated from a metastatic site in the lymph node of a 51 year old Caucasian male. Like HCT116, SW620 cells harbour an activating KRAS mutation (KRAS^G12V^) as well as heterozygous mutations in TP53 (TP53^R273H^ and TP53^P309S^) and homozygous mutation of APC (APC^Q1338*^). Loss of IKKα or IKKβ delayed but did not abolish TNFα-driven phosphorylation and degradation of IκBα, consistent with both kinases being redundant for IκBα regulation (Figure 8A). IKKβ S177/181 phosphorylation was more persistent in IKKα KO cells relative to WT and the same was seen for IKKα S176/180 phosphorylation in the IKKβ KO clones (Figure 8A). TNFα-driven p65 S536 phosphorylation was unaffected by single IKK KO; phosphorylation of S468 was reduced in IKKα KO cells but unaffected by IKKβ KO. Activating phosphorylation of JNK1/2 in response to TNFα was enhanced and more persistent in both the IKKα or IKKβ clones; this was a notable difference to HCT116 cells where JNK activation was enhanced in IKKβ KO cells but not IKKα KO. p38 phosphorylation was largely unaffected by IKK loss. IKKα KO SW620 cells exhibited a far more modest (and non-significant) reduction in TNFα-induced NF-κB:Luc than was seen in HCT116 cells, whereas loss of IKKβ had no effect on NF-κB:Luc (Figure 8B).

**Figure 9.**
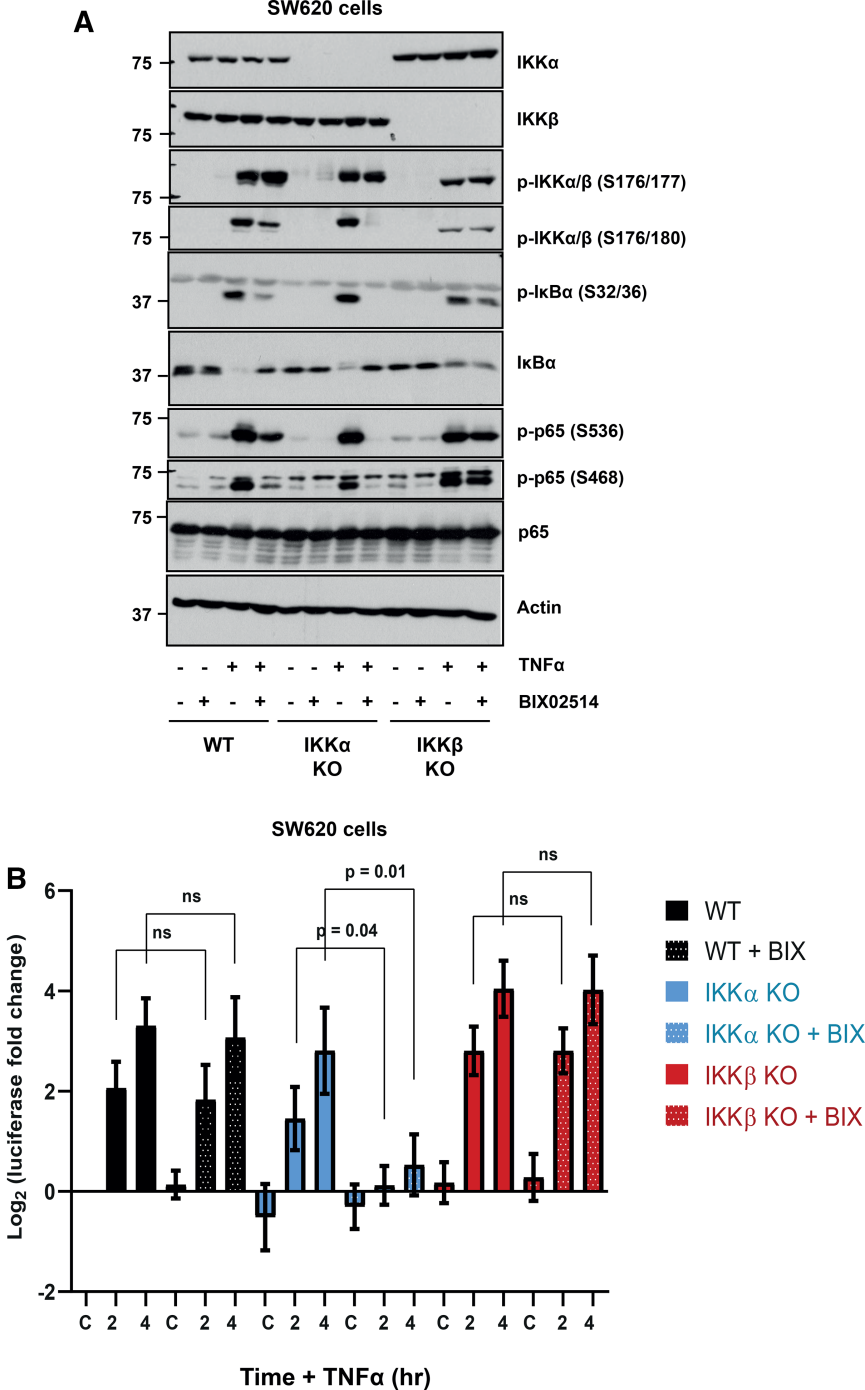
Selective IKKβ inhibition confirms a prominent role for IKKα in canonical NF-κB signalling in SW620 cells. (**A**) WT, IKKα KO or IKKβ KO SW620 cells were seeded for 48 h prior to treatment with 10 µM BIX02514 or DMSO vehicle for 30 min before treating with TNFα (10 min) and processing for western blot. (**B**) WT, IKKα KO or IKKβ KO SW620 cells were transiently transfected with reporter plasmids DNA. The following day, cells were treated with BIX02514 or DMSO vehicle control for 30 min, prior to treatment with TNFα for 2 or 4 h. Firefly luciferase luminescence was normalised as above and expressed as log_2_(fold change in TNFα-induced luciferase activity relative to the relevant, matched untreated condition). Results are mean ± SD of three independent experiments, each seeded in technical triplicate. Significance testing was performed using two-way ANOVA (repeated measures) with Tukey post-hoc test. *P* values relate to comparison with corresponding timepoint(s) of WT control.

In WT cells a dose of BIX02514 (selective IKKβ inhibitor) that abolished NF-κB:Luc in IKKα KO cells had modest effects on NF-κB signalling and no effect on TNFα-induced NF-κB:Luc suggesting that loss of IKKβ activity was largely compensated for by IKKα (Figure 9A,B). In IKKα KO cells BIX02514 completely inhibited TNFα-induced IκBα phosphorylation, IκBα degradation, phosphorylation of p65 at S536 and S468 and abolished TNFα-induced NF-κB:Luc activity (Figure 9A,B). These results strongly suggest that the IKK activity observed in IKKα KO cells is due to IKKβ. BIX02514 had no effect on TNFα-induced canonical NF-κB signalling or NF-κB:Luc in IKKβ KO cells, confirming that the IKK activity observed in these cells is not due to residual IKKβ activity but rather IKKα.

## Discussion

One of the central dogmas of NF-κB signalling is that IKKβ is the dominant IKK in the canonical pathway, with IKKα playing a more important role in the non-canonical NF-κB activation pathway. However, the literature actually suggests that the role of IKKα and IKKβ varies with stimulus, cell and tissue type, developmental stage and species. We were interested in inflammatory signalling in colorectal cells so we generated IKK KOs in HCT116 and SW620 cells; our results indicate that both IKKα and IKKβ normally contribute to TNFα dependent NF-κB activation in SW620 cells whereas IKKα plays the dominant role in HCT116 cells.

Despite early reports of a non-redundant role for IKKβ in IκBα phosphorylation and turnover [43], our results are more consistent with later reports of redundant roles for IKKα and IKKβ [26,28] since loss of IKKα or IKKβ alone had little (SW620) or no (HCT116) effect on IκBα phosphorylation and turnover. However, our results provide clear evidence for a major role for IKKα in canonical NF-κB signalling. In HCT116 cells, IKKα was the dominant kinase involved in the basal p65 S536 phosphorylation, stimulated p65 S468 phosphorylation, nuclear translocation of p65 and the NF-κB-dependent transcriptional response to both TNFα and IL-1α. In these cells IKKβ homodimers were far less efficient at compensating for the loss of IKKα than IKKα homodimers were able to compensate for the loss of IKKβ; this was confirmed when siRNA was used to knock-down the nontargeted kinase in single KO cells. Critically, experiments using the selective IKKβ inhibitor BIX02514 confirmed that these observations were not specific to IKKα KO cells, and that IKKα also plays a significant role in these responses in WT cells. Some subtle differences were noted in SW620 cells; for example, BIX02514 reduced but did not abolish p65 phosphorylation in WT cells suggesting both IKKα and IKKβ were contributing to these responses.

Notably, the level of stimulus-induced activation loop phosphorylation of the remaining IKK subunit was enhanced in the IKKα or IKKβ KO cells. In both HCT116 and SW620 cells, IKKβ S177/181 phosphorylation was higher and more sustained in IKKα KO cells relative to WT and the same was seen for IKKα in the IKKβ KO clones. This higher level of phosphorylation did not reflect a compensatory increase in the expression of the remaining IKK subunit in the HCT116 cells, whereas we did see a consistent increase in IKKα in the IKKβ KO SW620 cells. The deubiquitylases CYLD and A20 cleave K63-linked polyubiquitin chains of signalling components upstream of IKK to terminate the NF-κB signalling response and A20 KO MEFs exhibit more sustained TNFα induced IKK activity. Interestingly, IKKα has been shown to play an important role in this feedback inhibition by phosphorylating the regulatory molecule, TAX1BP1 in response to TNFα/IL-1α; this promotes the assembly of the A20 ubiquitin-editing complex [46]. However, although A20 is induced upon NF-κB activation [47] we observed no increase in A20 abundance in response to TNFα until later time points (data not shown) so it is unlikely that reduced feedback inhibition explains the higher phosphorylation of the remaining IKK subunits. The enhanced phosphorylation of the remaining IKK subunit in IKK KO cells should be considered in the context of the current model for IKKβ activation which suggests that TAK1 phosphorylation of IKKβ at Serine 177 primes the subsequent autophosphorylation of IKKβ on Serine 181 [48]. Our observations with an IKKβ selective inhibitor are consistent with this study. The enhanced IKKβ-dependent autophosphorylation at Serine 181 in IKKα KO cells and IKKα S180 phosphorylation in the IKKβ KO clones suggests that each IKK is more active in the absence of the other, or more readily activated by TAK1 in the absence of the other (i.e. a stoichiometry or competition effect). Clearly, further work is required to assess the kinetics of TAK1 activation and feedback control [49] in the IKK KO cells.

Serine 468 of p65 has been proposed as an IKKβ-dependent phosphorylation site since recombinant IKKβ phosphorylated this site more efficiently than IKKα *in vitro* [43], whilst dominant negative IKKβ, but not an equivalent IKKα construct, inhibited the TNFα and IL-1α induced phosphorylation of this site. However, we observed a much greater defect in the TNFα and IL-1α induced S468 phosphorylation in IKKα KO cells compared with IKKβ KO cells, suggesting that IKKα homodimers were better able to compensate for the loss of IKKβ in terms of S468 phosphorylation than IKKβ homodimers were for the loss of IKKα. The minimal role for IKKβ in S468 phosphorylation in WT HCT116 cells in response to TNFα was also demonstrated with the IKKβ inhibitor BIX02514. Unfortunately, although knockout and siRNA-mediated knockdown of IKKα interfered with the phosphorylation of this site, we were unable to convincingly demonstrate sufficiency IKKα re-expression. This could reflect a technical limitation of the transient transfections or transiently transfected FLAG-tagged IKKα and endogenous IKKα may not be functionally equivalent. Alternatively, IKKα may facilitate recruitment of another kinase such as GSK3β and IKKε [48,49] to phosphorylate S468 in response to TNFα. First-in-class IKKα selective inhibitors have recently been described [50] and may be commercially available in the near future. Such inhibitors would be valuable in resolving the importance of IKKα in S468 phosphorylation in response to TNFα and IL-1α. It will also be interesting to characterise the function of S468 phosphorylation in more detail in these cells. S468 phosphorylation by IKKβ occurs in the cytosol while p65 is bound to IκBα and appears to moderately inhibit TNFα and IL-1β induced NF-κB activation of certain genes [43]. This has been proposed to be due to S468 phosphorylation enhancing binding of p65 to the histone deacetylase, GCN5, which in turn recruits a COMMD1-containing E3 ligase complex that targets p65 bound to specific genes for proteasomal degradation [51,52]; thus the reduced S468 phosphorylation may not account for the reduced nuclear translocation of p65 in IKKα KO cells. Clearly, further work is required to resolve the function of S468 phosphorylation.

The marked reduction in TNFα- or IL-1α-induced nuclear translocation of p65 in IKKα KO HCT116 cells is likely to contribute to the lower NF-κB transcriptional activation in these cells. It is also notable that in the same cells TNFα-induced nuclear translocation of c-Rel was equally dependent on IKKα and IKKβ and these results were confirmed independently by sub-cellular fractionation. Thus, different NF-κB subunits exhibit different dependencies on IKKα or IKKβ for their nuclear translocation and these differences cannot be due to differences in IκBα degradation where individual IKK KO has little or no effect. It seems more likely that they are due to differences in IKK-dependent phosphorylation and potentially the sub-cellular location of the IKKs. IKKα has been proposed to have nuclear-specific roles in both the enhancement and termination of NF-κB-dependent gene expression, such as the phosphorylation of Histone H3 and CBP at the promoters of specific genes. The contributions of these proposed nuclear functions of IKKα to the observations made here have yet to be explored. However, other factors could influence the lower NF-κB transcriptional activation observed, and future studies should seek to define the relative DNA binding activities of different NF-κB dimers in IKKα and IKKβ KO cells and assess promoter occupancy of different NF-κB dimers on specific genes using chromatin immunoprecipitation (ChIP). Furthermore, since the IKKα KO cells exhibit reduced p65 S468 phosphorylation, which has been suggested to promote the proteolytic removal of p65 from the promoters of certain genes, it would be interesting to define the kinetics of promoter occupancy in these cells relative to WT; the overall level of p65-driven expression of certain genes might be lower but perhaps more sustained in these cells for this reason.

One final issue is the role of NEMO and NIK in the single IKK KO cells that we have derived, especially the IKKβ null cells. It has previously been shown that IKKα-dependent activation of the non-canonical NF-κB pathway requires NIK but is independent of NEMO [53]. It will be interesting to see if the IKKα-dependent NF-κB activation we report here is NEMO-independent and represents NIK stabilisation, and compensatory activation of non-canonical IKK signalling that feeds into the canonical pathway to compensate for loss of IKKβ. However, the non-canonical pathway is typically slower to elaborate as it requires NIK stabilisation; for example, we see no evidence of NIK stabilisation in IKKβ KO cells until 24 h after TNFα stimulation (data not shown), suggesting that this may not explain our NF-κB functional reporter assays which were all confined to 2, 4 or 6 h stimulation. These questions will require further investigation.

In summary, this is to our knowledge the first report of IKKα KO, IKKβ KO and IKKα/β DKO in human cells. Our results challenge the prevailing dogma by showing that IKKα is equally (SW620 cells) or more (HCT116 cells) important than IKKβ for mediating the activation of canonical NF-κB signalling in response to TNFα and IL-1α in colorectal cells. These results were confirmed by the use of selective IKK siRNA and IKKβ-selective small-molecule inhibition and should refocus efforts towards the development of IKKα selective inhibitors [50]. These IKK KO cells may prove valuable for future studies into NF-κB-dependent and NF-κB-independent functions of IKKα and IKKβ.

## Data Availability

All relevant data are contained within the main article and its Supplementary Files.

## Abbreviations

CAC: colitis-associated cancer
CRC: colorectal cancer
DKO: double knockout
IBD: inflammatory bowel disease
IKKβ: Inhibitor of kappa B kinase β
KD: kinase dead
MEFs: mouse embryonic fibroblasts
PI: propidium iodide
TNF: tumour necrosis factor
