# Supplementary material for: IKKα plays a major role in canonical NF-κB signalling in colorectal cells
Source: Biochem J. 2022 Feb 4;479(3):305–25. doi: 10.1042/BCJ20210783 (PMC8883499; doi:10.1042/BCJ20210783)

**Supplementary Figure Legends**

**Supplementary Figure 1. Characterisation of CRISPR-Cas9 IKKα knockout in HCT116 cells by Sanger sequencing.** Sanger sequencing screen shots with annotation for representative clones for IKKα in IKKα single KO clone F6 (**A**) and IKKα in IKKα/β DKO clone C8 (**B**) In each case the KO or DKO clone sequence is referenced to a control WT clone (clone A3) and screenshots are annotated with the position of the guide sequence and predicted cut site. Impact on reading frame and predicted translation product are also shown and are consistent with our immunoblots (with both N-term and C-term antibodies), which show complete KO of IKKα (Supplemental Figure 3).

**Supplementary Figure 2. Characterisation of CRISPR-Cas9 IKKβ knockout in HCT116 cells by Sanger sequencing.** Sanger sequencing screen shots with annotation for representative clones for IKKβ in IKKβ single KO clone G9 (**A**) and IKKβ in IKKα/β DKO clone C8 (**B**) In each case the KO or DKO clone sequence is referenced to a control WT clone (clone A3) and screenshots are annotated with the position of the guide sequence and predicted cut site. Impact on reading frame and predicted translation product are also shown and are consistent with our immunoblots (with both N-term and C-term antibodies), which show complete KO of IKKβ (Supplemental Figure 3).

**Supplementary Figure 3. Characterisation of CRISPR-Cas9 IKK knockout HCT116 cells by Western immunoblot.**

Representative WT, IKKα KO, IKKβ KO and IKKα/β DKO HCT116 cells were seeded in normal growth medium for 48 hours prior to lysis in TG lysis buffer (T) or RIPA buffer (R). Lysates were fractioned by SDS-PAGE and Western blotted with antibodies raised to the N- or C-termini of IKKα or IKKβ; the whole blots are shown to confirm absence of full length IKK protein and any truncated forms. Data are from a single experiment representative of two showing similar results. *Probable truncated forms of IKKα.

**Supplementary Figure 4. Characterisation of CRISPR-Cas9 IKK knockout HCT116 cells by RT-qPCR.**

(**A and B**) Three independently derived WT clones and three clones each of IKKα single, IKKβ single and IKKα/β double knockout were seeded in normal growth medium for 48 hours prior to RNA extraction. Relative IKKα (**A**) and IKKβ (**B**) mRNA expression was determined by RT-qPCR, with normalisation to the geometric mean of the reference gene (YWHAZ and UBC) expression. Expression ratios are plotted on a logarithmic scale as median IKK expression

ratios relative to the WT control samples. Boxes represent the interquartile range. Whiskers represent the minimum and maximum observations.

**Supplementary Figure 5. IKKα or IKKβ KO enhances and sustains the activation of the remaining IKK. (A**) WT (A3) and IKKα KO (F6) or (B) WT (A3) and IKKβ KO (G9) HCT116 cells were seeded in normal growth medium for 24 hours, prior to treatment with 10 ng/mL recombinant TNFα for the indicated time points. Whole cell extracts were prepared, fractionated by SDS-PAGE and Western blotted with the indicated antibodies. p-, phospho-.

**Supplementary Figure 6. Re-expressing WT but not kinase-dead IKK restores NF-κB activity to IKK KO HCT116 cells. (A)** WT (A3), IKKα KO (F6), IKKβ KO (G9) and IKKα/β DKO (C8) HCT116 cells were transiently transfected with empty vector (EV), WT IKKα (WTα), KD IKKα (KDα), WT IKKβ (WTβ) and KD IKKβ (KDβ), NF-κB firefly luciferase and renilla luciferase plasmids. The following day, cells were treated with 10 ng/mL TNFα (4 hours). Firefly luciferase was normalised to renilla and data expressed as relative luciferase activity (luciferase/renilla activity), RLU (relative light units). Results are mean ± SD of two independent experiments performed in technical triplicate. **(B)** WT and IKKα/β DKO HCT116 cells were transiently transfected with empty vector (EV), pCMV-Tag2B-WT IKKα, pCMV-Tag2B-KD IKKα (K44A), pCMV-Tag2B-WT IKKβ or pCMV-Tag2B-KD IKKβ (K44M). 24 hours later, cells were treated with 10 ng/mL TNFα for 5 or 10 minutes and whole-cell lysates fractioned by SDS-PAGE and Western blotted with the indicated antibodies. Data are from a single experiment representative of two showing similar results. **(C)** WT and IKKα KO HCT116 cells were transiently transfected with empty vector (EV), pCMV-Tag2B-WT IKKα or pCMV-Tag2B-KD IKKα (K44A). 24 hours later, cells were treated with 10 ng/mL TNFα for 10 or 30 minutes. Whole-cell lysates were fractioned by SDS-PAGE and Western blotted with the indicated antibodies as in (B). Data are from a single experiment representative of two showing similar results.

**Supplementary Figure 7. IKKα KO and IKKβ KO have different effects on TNFα-induced nuclear translocation of p65 and c-Rel. (A)** WT and IKKα KO or **(B)** WT and IKKβ KO HCT116 cells were seeded in normal growth medium for 48 hours prior to treatment with 10 ng/mL TNFα for 10 or 30 minutes. Cytosolic (cyt) and nuclear (nuc) fractions were prepared and Western blotted with the indicated antibodies. β-tubulin and Lamin A/C were used as markers of the cytosol and nucleus, respectively. p-, phospho- *unknown band. **(C)** WT and IKKα KO HCT116 cells were seeded in normal growth medium for 48 hours prior to treatment

with 10 ng/mL TNFα for 10 or 30 minutes. Cytosolic (cyt) and nuclear (nuc) fractions were prepared and Western blotted with the indicated antibodies. **(D)** WT and IKKα KO HCT116 cells were seeded prior to treatment of WT cells with 20 nM siRNA targeted against IKKα. Immunofluorescence staining of cells was performed with anti-IKKα (N-terminal Ab) (red) and nuclei with DAPI (blue). Scale bar, 10 μm. Data are from a single experiment representative of two showing similar results.

**Supplemental Figure 1**

# A

## IKKα KO – KO Clone F6 compared with WT clone A3 (IKKα gene)

### Allele 1 – Large deletion across exon 1-2 boundary

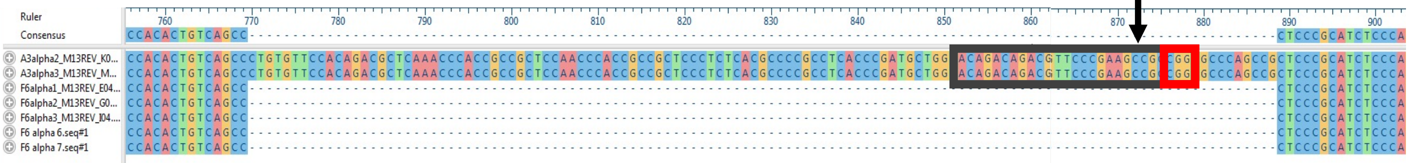

### Allele 2 – Single nt deletion – premature stop codon after first 37 aa

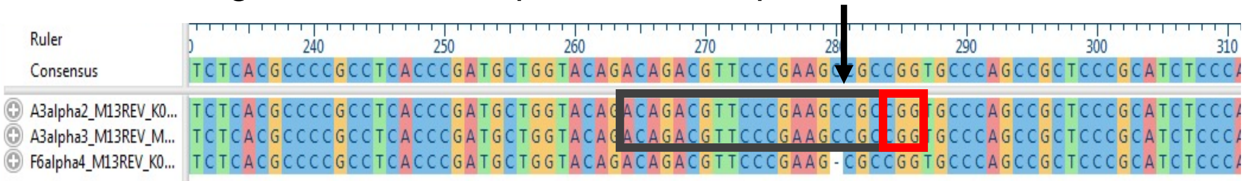


### 5'3' Frame 1 – premature stop codon after 37 aa

M E R P P G L R P G A G G P W E M R E R L G T G A S G T S V C T S I G N L I S K Stop Q L S L V A Stop S Stop V P K T E N D G A M K S R L Stop R S Stop T M P M L Stop R
P V M F L K N Stop I F Stop F M M C L F Stop Q W N T V L E E I S E S C S T N Q K I V V D L K K A R Y F L Y Stop V I Stop G L G F D I C M K T K L Y I E I Stop N L K T Stop F F
R M L V E R Stop Y I K Stop L I W D M P K M L I K E V C V H L L W E H C S I W P Q S S L R I S L T Q P L L I I G A L G P W Y L N V L L D I G L F C I I C S H L P G M R R L R R R
I Q S V Y L H V K R C Q E K F G L V A I Y L N Q I A F V V Stop _ Stop N P W K T G Y S Stop C Stop I G T L S R E E D L L T L L Stop S S Q D V L Y Stop W I T F Stop I Stop R
Stop Y T S Stop I Stop L L Q R Stop F L F C Y H L M K V F I H Y S L V L S V K L E Stop I L V L K N F F Q R Q E F L W I L G N Q P L N V F Stop M E L E A V I A I W F I C L I K V
K L Y M K G H L L P E V Y L I V Stop I I L Y R T A K Y S F Q L Y S C V K C G L K Q C T M C L D Stop K K T I A G S F R D K G Q Q C Stop V F L D I M L T Stop Q K Stop R T L
Stop S Q H H N N Stop K L N W S F F T K A F S L T W R D T A S R Stop R M G Y L Q K K C Stop K H G K K W K K R P S T M L R L V S L D T W R I R L C L C M L K S W S Y R
R A P M E D V R E T Stop W N L W N S V P L I Y I S S Stop N T D L Q I T P T V T A Q R W Stop K S L C T L C R V R T V C S R S C L V I Stop A S C W A V S R R L L I Y S L R W
K W P S V I S K K L T I L S C S C R E K G R K K Y G I S L K L P V H R V L P G P L Stop D P V Stop K V Q Stop P L R H Q H G C P R L Q Q N M I I L C H V W Stop L L K M G R L
Q H K Stop _ K K I Stop T A L A I Stop A L L F M R Q M R N R A I V Stop _ I L I G V G Stop Q N

---

# B

## IKKα/IKKβ DKO – DKO Clone C8 compared to WT clone A3 (IKKα gene)

### Allele 1 – Large deletion

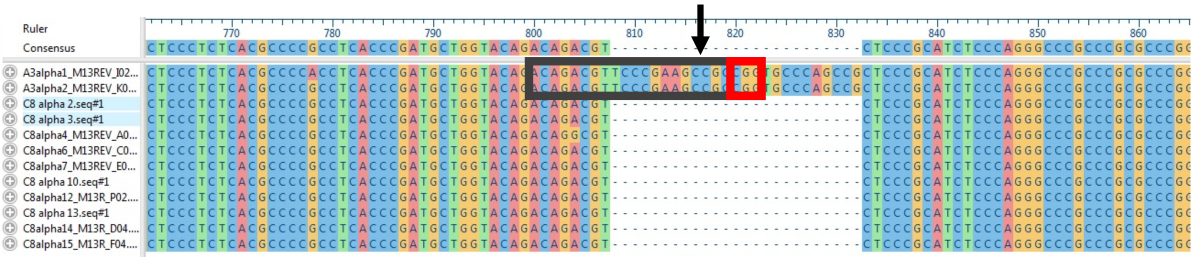

### 5'3' Frame 1 - Premature stop codon after 37 aa

M E R P P G L R P G A G G P W E M R E T S V C T S I G N L I S K Stop Q L S L V A Stop S Stop V P K T E N D G A M K S R L Stop R S Stop T M P M L Stop R P V M F L K N
Stop I F Stop F M M C L F Stop Q W N T V L E E I S E S C S T N Q K I V V D L K K A R Y F L Y Stop V I Stop G L G F D I C M K T K L Y I E I Stop N L K T Stop F F R M L V E R
Stop Y I K Stop L I W D M P K M L I K E V C V H L L W E H C S I W P Q S S L R I S L T Q P L L I I G A L G P W Y L N V L L D I G L F C I I C S H L P G M R R L R R R I Q S V Y L
H V K R C Q E K F G L V A I Y L N Q I A F V V Stop _ Stop N P W K T G Y S Stop C Stop I G T L S R E E D L L T L L Stop S S Q D V L Y Stop W I T F Stop I Stop R Stop Y T S
Stop I Stop L L Q R Stop F L F C Y H L M K V F I H Y S L V L S V K L E Stop I L V L K N F F Q R Q E F L W I L G N Q P L N V F Stop M E L E A V I A I W F I C L I K V K L Y M K G
H L L P E V Y L I V Stop I I L Y R T A K Y S F Q L Y S C V K C G L K Q C T M C L D Stop K K T I A G S F R D K G Q Q C Stop V F L D I M L T Stop Q K Stop R T L Stop S Q H H
N N Stop K L N W S F F T K A F S L T W R D T A S R Stop R M G Y L Q K K C Stop K H G K K W K K R P S T M L R L V S L D T W R I R L C L C M L K S W S Y R R A P M E D V
R E T Stop W N L W N S V P L I Y I S S Stop N T D L Q I T P T V T A Q R W Stop K S L C T L C R V R T V C S R S C L V I Stop A S C W A V S R R L L I Y S L R W K W P S V I S
K K L T I L S C S C R E K G R K K Y G I S L K L P V H R V L P G P L Stop D P V Stop K V Q Stop P L R H Q H G C P R L Q Q N M I I L C H V W Stop L L K M G R L Q H K Stop _
K K I Stop T A L A I Stop A L L F M R Q M R N R A I V Stop _ I L I G V G Stop Q N

# Supplemental Figure 2

## A

### IKKβ KO – KO Clone G9 compared with WT clone A3 (IKKβ gene)

**Allele 1** – Deletion of 2 nt

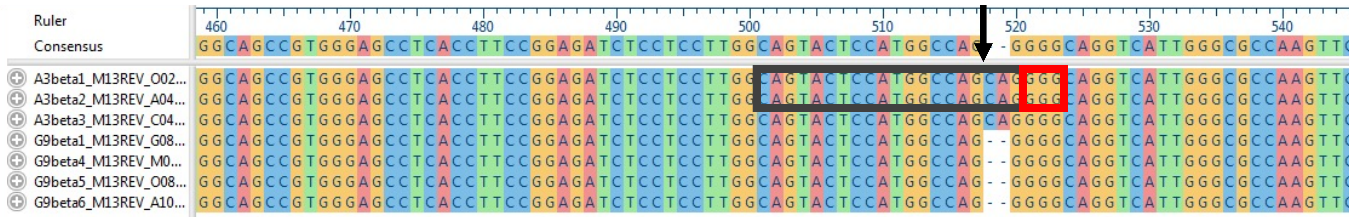


5'3' Frame 1 - Premature stop codon after 110 aa

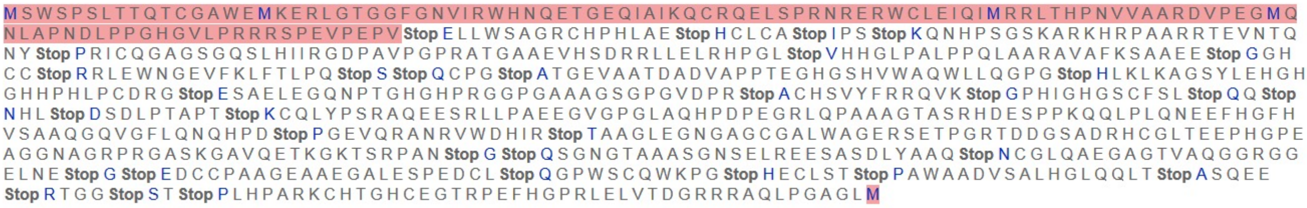

**Allele 2** – Substitution

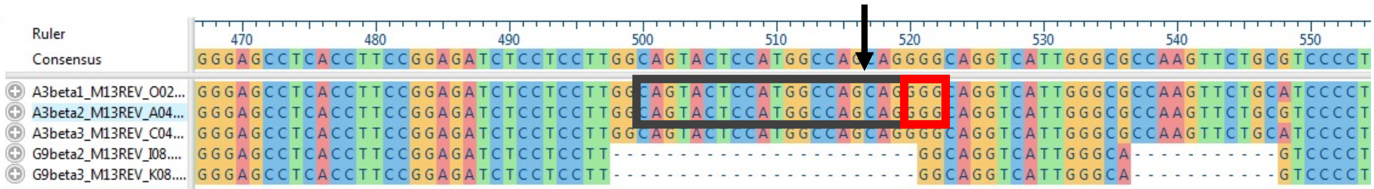


5'3' Frame 1 - Premature stop codon after 85 aa

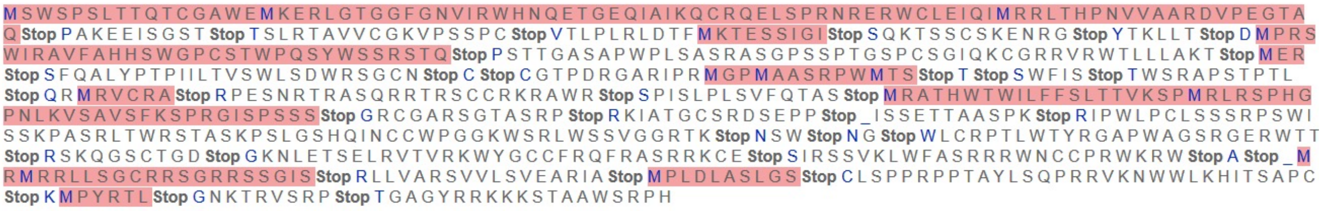

---

## B

### IKKα/β DKO – DKO Clone C8 compared to WT clone A3 (IKKβ gene)

Deletion of a single nt

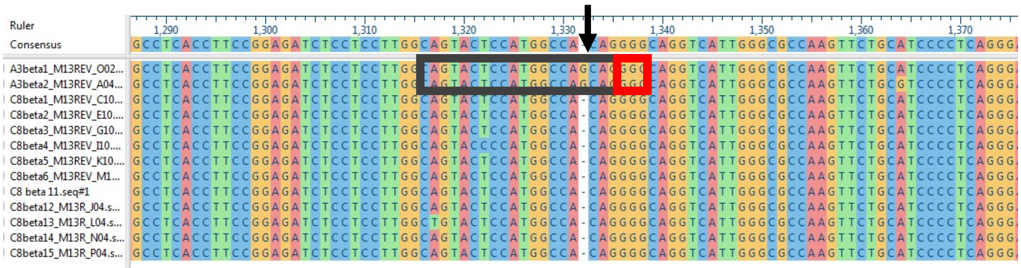


5'3' Frame 1 - Premature stop codon after 107 aa

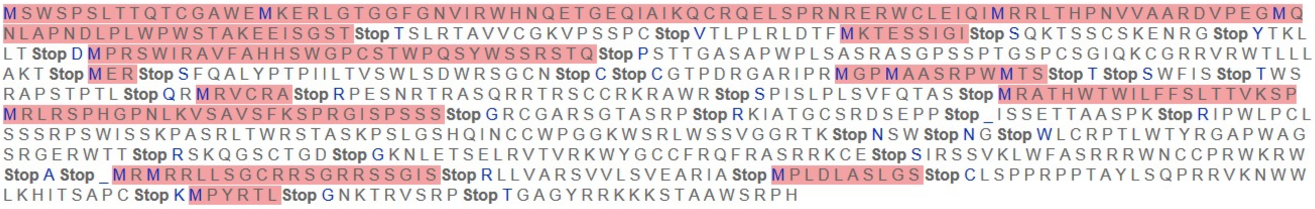

**Supplemental Figure 3**

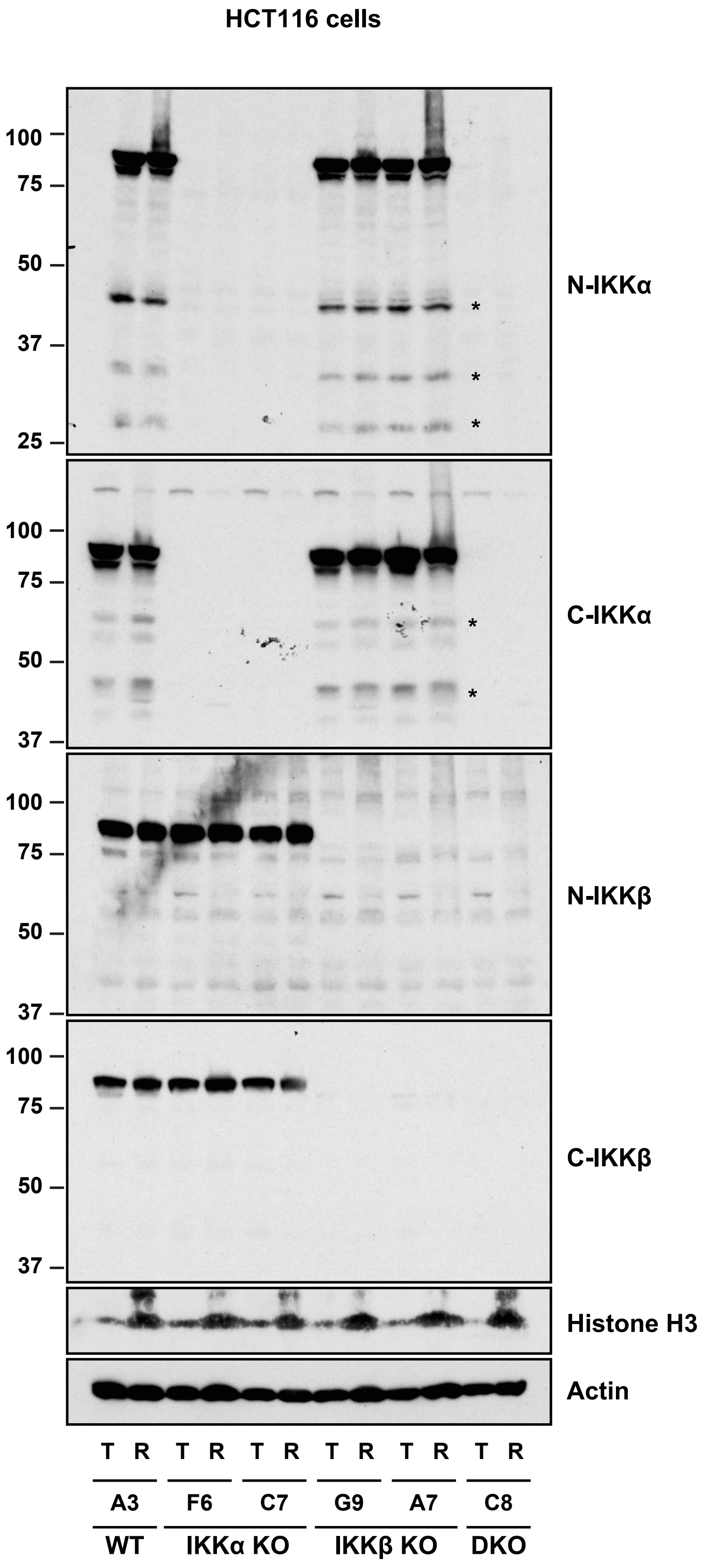

**Supplemental Figure 4** **HCT116 cells**

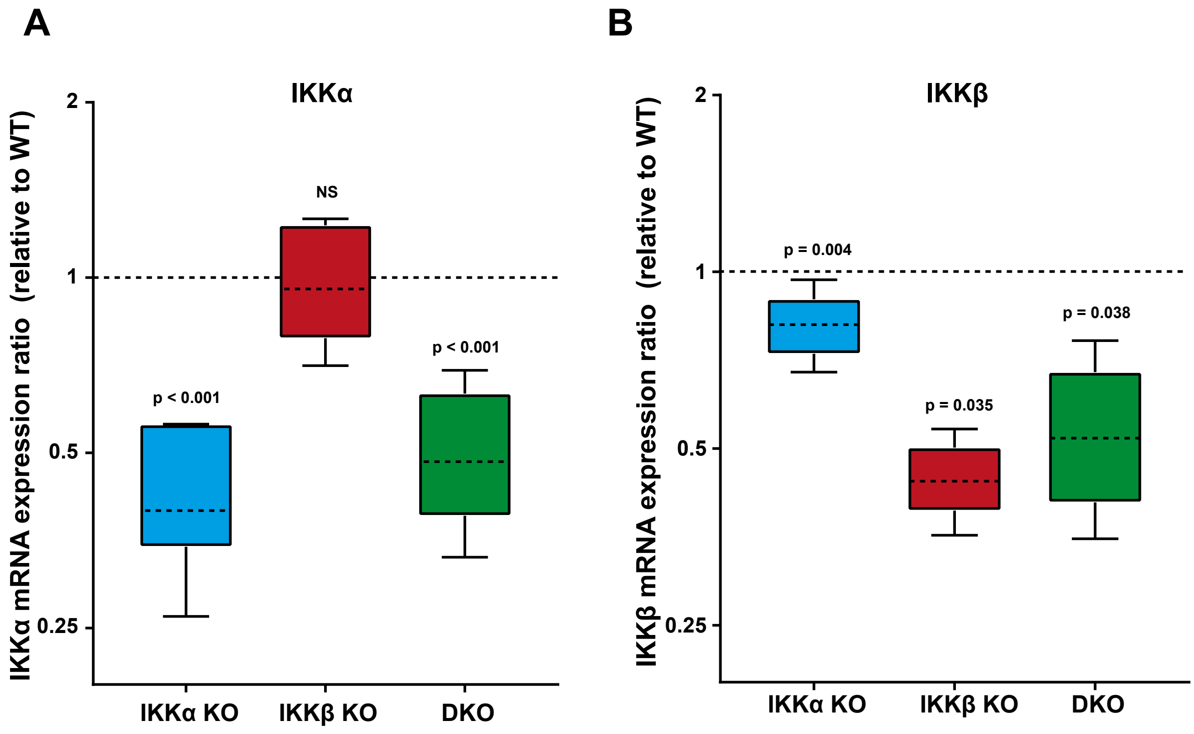

**Supplemental Figure 5**

**HCT116 cells**

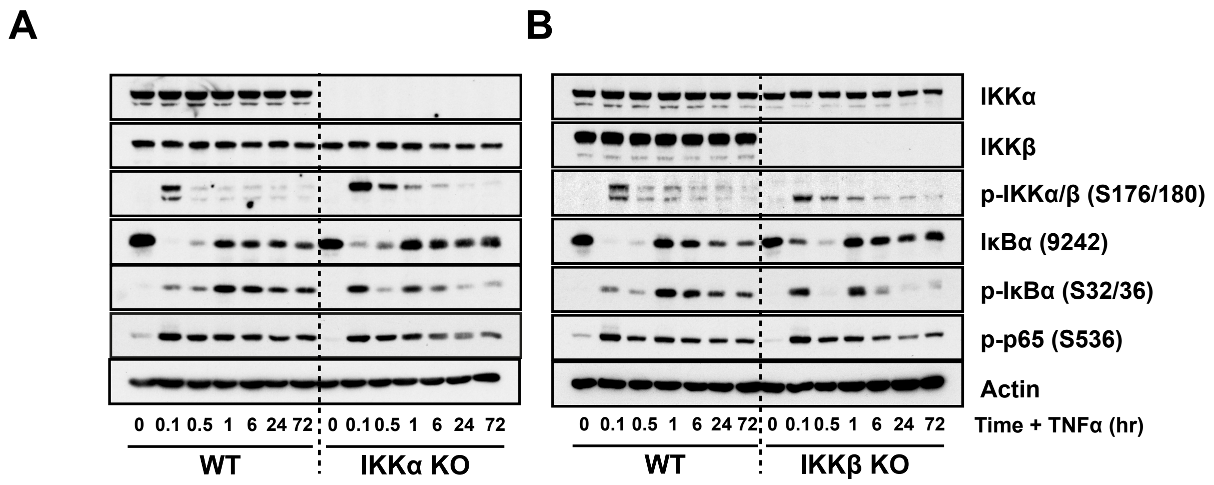

Supplemental Figure 6

HCT116 cells

A

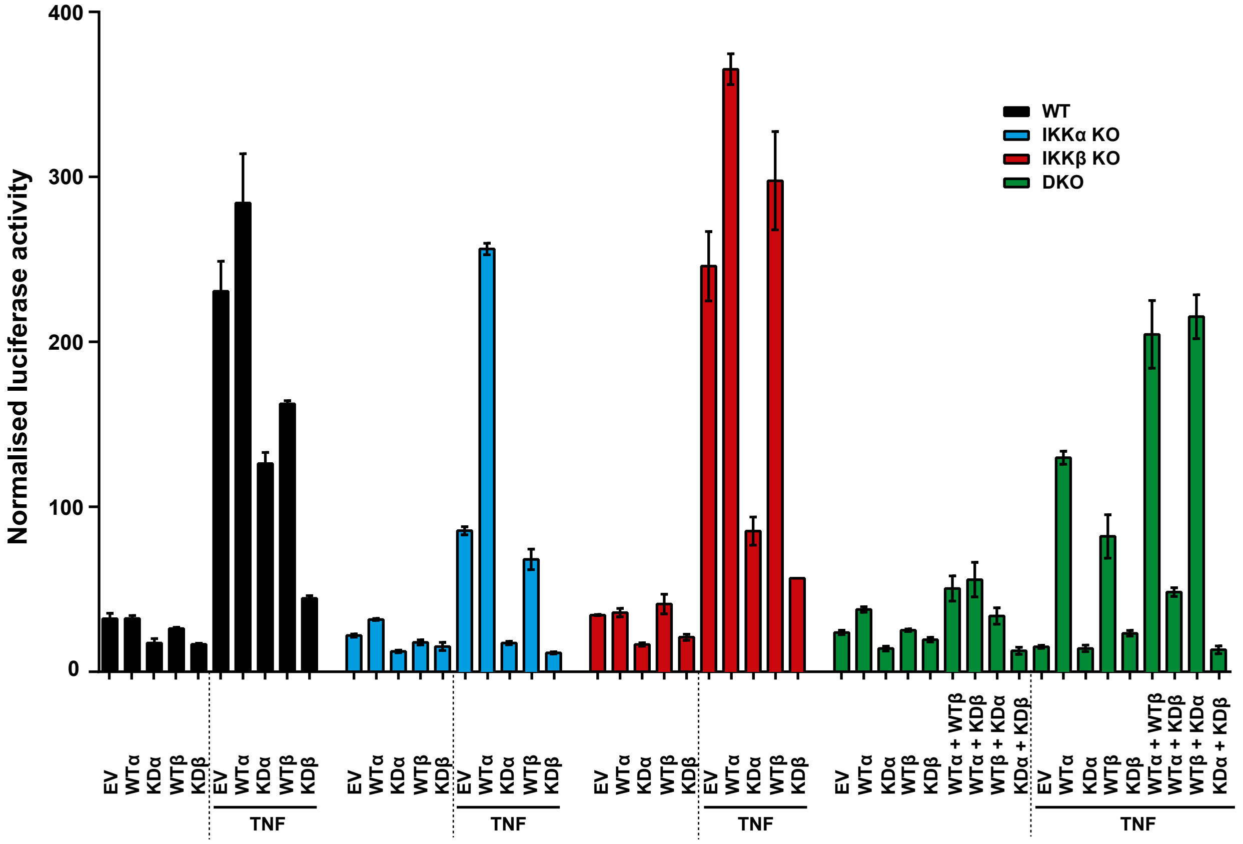


B

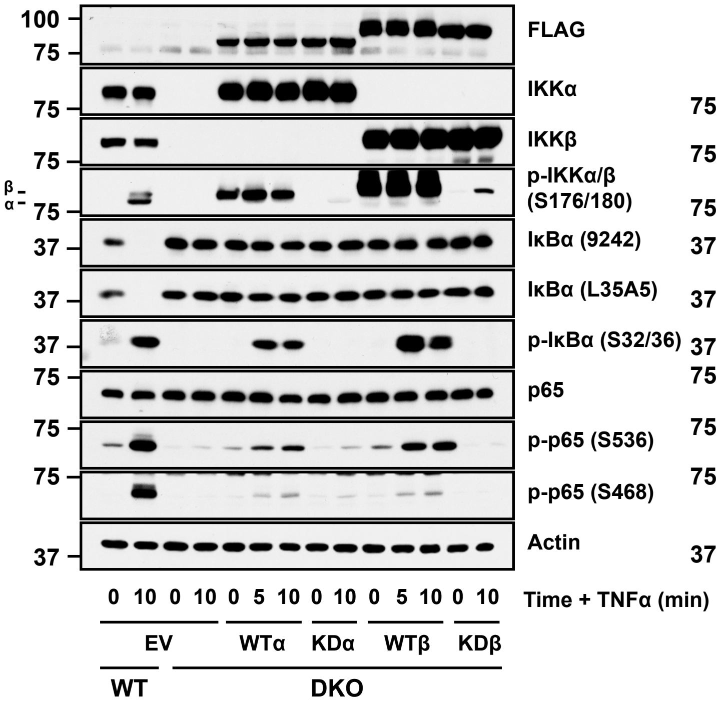


C

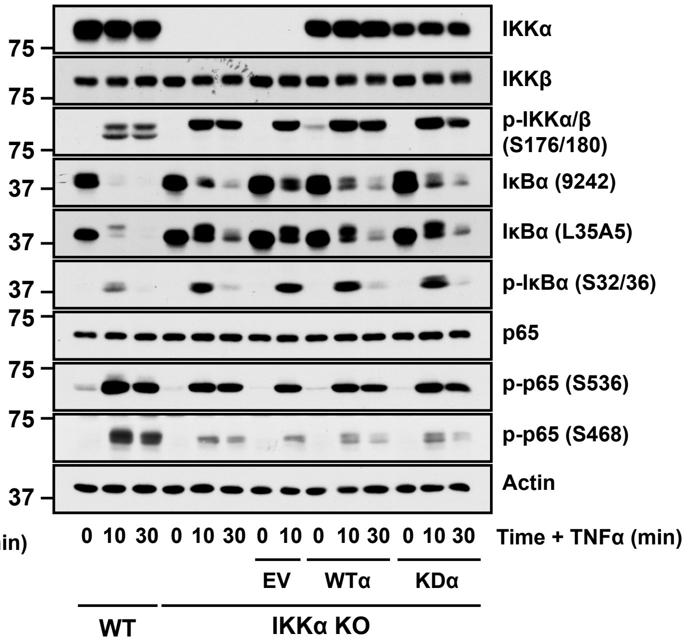

Supplemental Figure 7 HCT116 cells

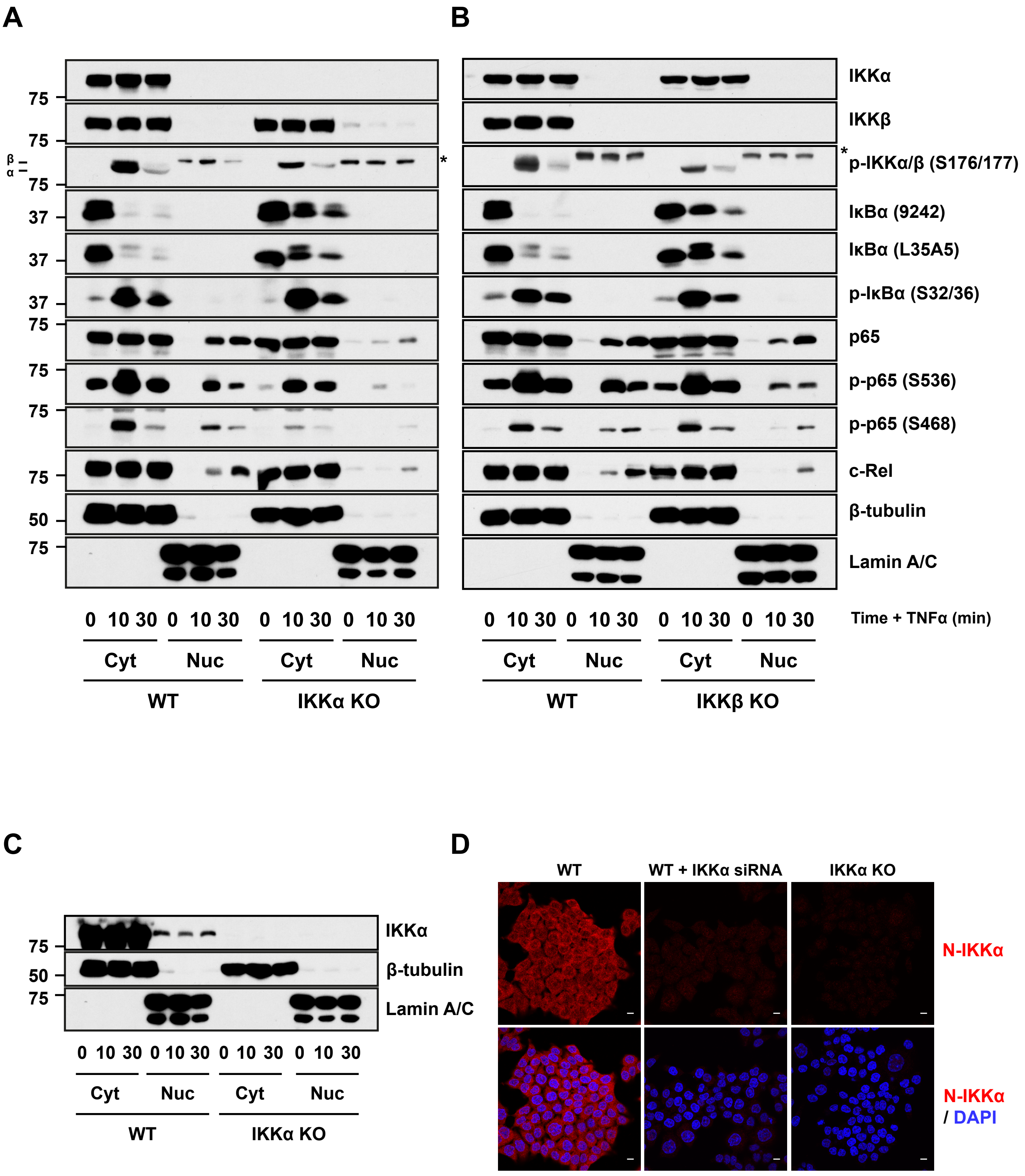

Supplement: Supplementary Material [file BCJ-479-305-s1.pdf]
